# Transforming growth factor-β1 protects against LPC-induced cognitive deficit by attenuating pyroptosis of microglia via NF-κB/ERK1/2 pathways

**DOI:** 10.1186/s12974-022-02557-0

**Published:** 2022-07-28

**Authors:** Yi Xie, Xuejiao Chen, Ying Li, Simiao Chen, Shuai Liu, Zhiyuan Yu, Wei Wang

**Affiliations:** 1 Department of Neurology, Tongji Hospital, Tongji Medical College, Huazhong University of Science and Technology, Wuhan, 430030 China; 2 Department of Rehabilitation Medicine, The First Affiliated Hospital of Medical College, Zhejiang University, Hangzhou, 310003 China; 3 Reproductive Medicine Center, Tongji Hospital, Tongji Medicine College, Huazhong University of Science and Technology, Wuhan, 430030 China

**Keywords:** Demyelination, Cognitive deficit, LPC, TGF-β1, Microglia, Pyroptosis, Neuroinflammation

## Abstract

**Background:**

Demyelinating diseases in central nervous system (CNS) are a group of diseases characterized by myelin damage or myelin loss. Transforming growth factor beta1 (TGF-β1) is widely recognized as an anti-inflammatory cytokine, which can be produced by both glial and neuronal cells in CNS. However, the effects of TGF-β1 on demyelinating diseases and its underlying mechanisms have not been well investigated.

**Methods:**

A demyelinating mouse model using two-point injection of lysophosphatidylcholine (LPC) to the corpus callosum in vivo was established. Exogenous TGF-β1 was delivered to the lesion via brain stereotactic injection. LFB staining, immunofluorescence, and Western blot were applied to examine the severity of demyelination and pyroptosis process in microglia. Morris water maze test was used to assess the cognitive abilities of experimental mice. Furthermore, lipopolysaccharide (LPS) was applied to induce pyroptosis in primary cultured microglia in vitro, to explore potential molecular mechanism.

**Results:**

The degree of demyelination in LPC-modeling mice was found improved with supplement of TGF-β1. Besides, TGF-β1 treatment evidently ameliorated the activated proinflammatory pyroptosis of microglia, with downregulated levels of the key pyroptosis effector Gasdermin D (GSDMD), inflammasomes, and cleaved-IL-1β, which effectively attenuated neuroinflammation in vivo. Evaluated by behavioral tests, the cognitive deficit in LPC-modeling mice was found mitigated with application of TGF-β1. Mechanistically, TGF-β1 could reverse pyroptosis-like morphology in LPS-stimulated primary cultured microglia observed by scanning electron microscopy, as well as decrease the protein levels of cleaved-GSDMD, inflammasomes, and cleaved-IL-1β. Activation of ERK1/2 and NF-κB pathways largely abolished the protective effects of TGF-β1, which indicated that TGF-β1 alleviated the pyroptosis possibly via regulating NF-κB/ERK1/2 signal pathways.

**Conclusions:**

Our studies demonstrated TGF-β1 notably relieved the demyelinating injury and cognitive disorder in LPC-modeling mice, by attenuating the inflammatory pyroptosis of microglia via ERK1/2 and NF-κB pathways. Targeting TGF-β1 activity might serve as a promising therapeutic strategy in demyelinating diseases.

**Supplementary Information:**

The online version contains supplementary material available at 10.1186/s12974-022-02557-0.

## Background

Demyelinating lesion in central nervous system (CNS) is a pathological process characterized by a loss of myelin sheaths surrounding axons, which is responsible for multiple sclerosis (MS), neuromyelitis optica (NMO), acute disseminated encephalomyelitis (ADEM), etc. [1, 2]. Demyelination leads to impaired transmission of nerve impulses, accompanied by neurodegeneration with impairment of spatial learning and memory [3, 4]. The potential causes of demyelination involve inflammation, hypoxic ischemia, viral infection, and autoimmune attack [5]. However, the underlying cellular and molecular mechanisms of demyelination are poorly understood. A commonly used rodent model of toxin-induced demyelination is stereotaxic injection of concentrated lysophosphatidylcholine (LPC) into CNS [6]. Two-point injection of LPC could induce a focal demyelinating lesion that peaks in size about 24 to 72 h later and remains in a long manner, which reproduces key features of demyelination in MS [6, 7].

Neuroinflammation is generally involved in the pathology of inflammatory demyelinating disease, such as MS [8]. Microglia, the major innate immune cells in CNS, become activated and play a prominent role in neuroinflammation [9]. Many evidences point to the neurotoxic nature of microglia response [10, 11], whereas some others indicate that microglia-mediated neuroinflammation is possibly beneficial in certain circumstances, such as stimulating myelin repair, removing toxic aggregated proteins and cell debris from CNS [12–14]. Pyroptosis, an inflammatory form of programmed cell death, relies on the activity of inflammasomes and cytosolic Gasdermin D (GSDMD) to release proinflammatory cytokines including IL-1β and IL-18 [15]. NLRP3 inflammasome, so far the most commonly studied and the best characterized inflammasome in microglia [16], is a multiprotein complex of NLRP3, apoptosis-associated speck-like protein (ASC) and caspase-1, which mediates the activation of caspase-1 and subsequently promotes the maturation and release of IL-1β and IL-18 [17]. Recent studies demonstrated that microglia-associated pyroptosis played an important part in the inflammatory processes in multiple rodent models of CNS injuries, such as spinal cord injury [15] and traumatic brain injury [18]. Other studies have also reported increased expression of inflammasome components in CNS tissues from MS patients [19, 20].

Transforming growth factor betas (TGF-βs), known as multifunctional growth factors, participate in the regulation of key events of development, disease and tissue repair [21]. TGF-βs family is represented by three isoforms: TGF-β1, -β2 and -β3, all of which are produced by glial and neuronal cells in central nervous system [22]. Among them, it was reported that administration of TGF-β1 could significantly reduce acute neuronal loss in modeling-rodents of hypoxic-ischemic (HI) brain injury [23], and prevent disease severity in experimental autoimmune encephalomyelitis (EAE) [24]. Moreover, the expression level of TGF-β1 in serum obtained from the patients with Alzheimer’s disease (AD) is evidently lower when compared with healthy controls, and there is a negative correlation (*p* < 0.05) between the serum TGF-β1 levels and the Clinical Dementia Rating (CDR) scores in patients [25]. However, until now, the potential protective functions of TGF-β1 on demyelinating diseases and its molecular mechanisms are largely unknown and not examined in previous studies.

In this study, we proposed and verified that administration of TGF-β1 could evidently alleviate the demyelinating severity, microglial activation, and accompanied cognitive deficit in LPC-induced demyelinating mice model. Mechanistically, we further confirmed that treatment of TGF-β1 could suppress the pyroptosis of microglia both in vivo and in vitro, which led to reduced release of proinflammatory cytokines and ultimately promoted myelin sheath repair following demyelinating injury. Taken together, our research suggests that TGF-β1 is a potential therapeutic target for demyelinating diseases, via modulating microglial pyroptosis and neuroinflammation.

## Materials and methods

### Animals

All of the animal experiments were approved by the Institutional Animal Care and Use Committee of Tongji Hospital, Tongji Medical College, Huazhong University of Science and Technology (HUST). Efforts were made to minimize the number of animals used in this study. Adult wild‐type C57BL/6 J mice for in vivo experiments were obtained from the Vital River Laboratories, Beijing, China. Adult mice (20–25 g; 10–12 weeks old) were housed in a 12-h light/12-h dark cycle at the standard conditions of 22 °C temperature, with free access to water and food. Mice were randomly assigned to different experimental groups. P1–P3 neonatal C57BL/6 J mice pups for in vitro experiments were obtained from the Three Gorges University Laboratory Animal Center, Wuhan, China.

### Establishment of LPC-induced demyelinating model

The model of focal demyelination was established based on the research of Luo et al. with some modifications [6]. Mice were anesthetized with isoflurane (induced at 3%, and maintained at 1.5%), and mounted onto a stereotactic frame (RWD Life Science, China). Demyelination in corpus callosum was induced by stereotaxic injection of 2 μL of 1% LPC (Sigma, USA) in PBS at the rate of 0.5 μL/min using a 32-gauge needle attached to a 5-μL gas-tight glass syringe (Hamilton, USA). The first injection site was 1.0 mm lateral, 0.3 mm anterior to the bregma, and 2.0 mm deep. The second injection site was 1.0 mm lateral, 0.8 mm anterior to the bregma, and 2.2 mm deep. After injection, the needle was kept in each position for an additional 10 min to minimize backflow. Mice in Sham group were stereotaxically injected with equal volume of PBS at the same sites.

### TGF-β1 treatment in vivo

In TGF-β1-preventative experiments, two days prior to LPC modeling, 4 μL of 2 μg/mL TGF-β1 (Peprotech, USA) or PBS was stereotaxically injected into the lateral ventricle of mouse (1.0 mm lateral, 0.3 mm posterior to the bregma, and 2.7 mm deep) at the rate of 0.5 μL/min. On the modeling day, 2 μL of 1% LPC plus 1 μL of 2 μg/ml TGF-β or PBS were delivered to each modeling site at the rate of 0.5 μL/min using a 32-gauge needle attached to a 5-μL gas-tight glass syringe (Hamilton, USA). The modeling mice receiving TGF-β1 were termed as TGF-β1 group, while the modeling mice receiving PBS were termed as Vehicle group.

In TGF-β1-therapeutic experiments, the next day after LPC modeling, a guide cannula (C = 2.2 mm, RWD, China) was stereotaxically positioned into the lateral ventricle of mouse (1.0 mm lateral, 0.3 mm posterior to the bregma, and 2.2 mm deep). The guide cannula was then secured to the skull with dental cement. On the third day after LPC injection, 3.5 μL of 2 μg/mL TGF-β1 or PBS was delivered to the lateral ventricle through an injector cannula (G = 0.5 mm, RWD, China) fixed on a polyethylene (PE50) tube connected to a 25-μL syringe microinjector (Gaoge, China) at the rate of 0.3 μL/min. After each infusion, the injector cannula was remained in the guide cannula for 10 min to minimize backflow. The TGF-β1 treatments were conducted once a day for 7 consecutive days. The modeling mice receiving TGF-β1 were termed as TGF-β1 group, while the modeling mice receiving PBS were termed as Vehicle group.

### Primary cell culture and treatments

Primary cultured microglia were isolated from the brain of neonatal C57BL/6 J mice at P1-P3 as described with modifications [26, 27]. Briefly, the brain of neonatal mouse was separated with meninges removed, and then was minced into small pieces and digested with 0.125% trypsin for 10 min at 37 °C. The digested tissues were centrifuged at 1000 rpm for 10 min and suspended in high glucose DMEM medium (HyClone, USA) supplemented with 20% fetal bovine serum (FBS). The mixed glial cells were incubated at 37 °C in a 95% air and 5%CO_2_ incubator for 11–13 days, and the medium was changed every 3 days. Microglia were separated from the mixed glial cells by shaking the flasks at 200 rpm for 2 h at 37 °C. The obtained microglia cells were seeded onto plates at a density of 4 × 10^4^/cm^2^ in high glucose DMEM medium supplemented with 10% FBS for 24 h before further treatments.

Primary cultured microglia were respectively treated with LPS, LPS + TGF-β1, LPS + TGF-β1 + phorbol 12‑myristate 13‑acetate (PMA), and PBS for 24 h. LPS was used to induce pyroptosis of primary cultured microglia [15]. PMA was used as a strong agonist both for ERK1/2 and NF-κB signal pathways [28]. The applied dose was 1 μg/mL for LPS (sigma, USA), 10 ng/mL for TGF-β1 (Peprotech, USA), 1 μM for PMA (MedChem Express, China). The dose and duration of treatments were chosen based on our preliminary experiments and previous studies [15, 29].

### Morris water maze (MWM) test

The Morris water maze test is widely used to assess spatial learning and memory performance in rodents [30]. The apparatus consisted of a circular tank (150 cm in diameter and 40 cm in height) filled with warm water (20–23 °C). Four different pictures were hung on the curtain surrounding the tank in four quadrant directions to function as permanent distal cues. The water was made to appear opaque by the addition of powdered milk. A platform (10 cm in diameter) was located in the middle of southwest quadrant. Mice were subjected to four consecutive trials each day over a 5‐days training period. Each mouse was released in sequence from four different positions around the perimeter of tank (north, northwest, east, and southeast) in four trials [31]. In each trial, each mouse was allowed to swim until it found the platform (for a maximum of 60 s). If the platform was not found in 60 s, the mouse was guided to the platform and remained there for 15 s. The escape latency to find the hidden platform was automatically recorded using a video tracking system (XR, China). On the sixth day after 5-days training, a probe test was conducted. The platform was removed while each mouse was released from the northeast quadrant and allowed to swim for 60 s. Memory retention was measured by quantifying the time spent in the target quadrant (southwest) and the number of times crossing the previous platform location.

### TreadScan analysis

The motor ability of modeling mice was evaluated by TreadScan according to the protocol of a published study [32]. In brief, prior to the test day, mice were trained to walk on the motor-driven treadmill belt at a speed of 6 cm/s in 1 min for three times, during three consecutive days. On the test day, each mouse was allowed to walk on the treadmill belt at a speed of 6 cm/s for a period of 20 s. The foot-prints and body movement were recorded with a high-speed digital video camera from the ventral view of the treadmill belt reflected off the mirror. TreadScan software (Clever SYS, USA) was used to identify and analyze a number of gait-associated parameters, such as initial foot contact, stance duration, stride duration, swing duration, stride length, track width, and toe spread for each foot, etc. Subsequent bioinformatics analyses of the obtained data including principal component analysis (PCA) and clustering heatmap were performed using the R Programming Language. The R codes for PCA and heatmap were presented in Additional file 2.

### Tissue preparation

On the tenth day post modeling, mice were euthanized and transcardially perfused with 25 mL of 0.9% physiological saline. For immunofluorescence and histochemical staining, mice were then perfused with 25 mL of 4% paraformaldehyde (PFA). The fixed brains were removed and post-fixed in 4% PFA for 12 h (4 °C) and gradually dehydrated using 10%, 20%, and 30% sucrose in PBS. The sucrose solution was used to prevent brain from being damaged by the formation of water crystals during freezing process. Coronal brain slices of 12 μm were sectioned at -20 °C using a constant temperature freezing microtome (Thermo Fisher Scientific, USA). Slices were then gently mounted to the microslides and stored in − 80 °C for subsequent experiments. For Western blot, the injury focus and approximately 2 mm of surrounding tissue were isolated and rapidly frozen in cooled isopentane, and then stored in − 80 °C for further use.

### Luxol fast blue (LFB) staining

LFB staining was used to observe histological changes of myelin [33]. As instructed, the brain slice was incubated in LFB dye (Servicebio, China) at 60 ℃ for 8–10 h. After rinsed with PBS, the brain slices were differentiated alternately in a lithium carbonate solution and 70% ethanol, then dehydrated with 75%, 90%, and 100% ethanol. Finally, the slices were soaked in xylene for 5–10 min and sealed with neutral resin. Images were taken by an optical microscope (Olympus, Japan). The severity of myelin lesion in corpus callosum was graded as normal (grade 0), the disarrangement of nerve fibers (grade 1), the formation of marked vacuoles (grade 2), and the disappearance of myelinated fibers (grade 3) as described [33].

### Hematoxylin–eosin (HE) staining

HE staining was conducted according to the routine protocol [17]. In brief, sections were stained with hematoxylin solution (Servicebio, China) for 5 min followed by five dips in 1% acid ethanol (1% HCl in 70% ethanol), and then rinsed in distilled water. Subsequently, the sections were stained with eosin solution for 3 min followed by dehydration with graded alcohol and clearing in xylene. The stained slices were photographed and examined using an optical microscope (Olympus, Japan).

### Immunofluorescence (IF) and quantification

For immunofluorescent staining, frozen brain sections and 4% PFA-fixed cell climbing slices were washed by PBS for 5 min and then permeabilized by 0.3% Triton-X100 (Servicebio, China) in PBS for 15 min. After blocked with 10% bovine serum albumin (Servicebio, China) in PBS for 1 h, the samples were incubated with corresponding primary antibodies (Additional file 3) overnight at 4 °C and washed three times with PBS for 10 min. Then slices were incubated with secondary antibody (Invitrogen, USA) for 1 h in the dark at room temperature. After washed three times for 10 min each in PBS, the slices were mounted with an antifade mounting medium with DAPI (Beyotime, China). The sections were observed blindly under a fluorescence microscope (Olympus BX51, Japan) or a laser scanning confocal microscope (Olympus FV500, Japan). As LPC-modeling dramatically activated glial cells, the accumulation of DAPI^+^cells with obvious boundary was identified as the lesion. The area of total lesion and Iba1^+^cells were quantified using ImageJ software (National Institutes of Health, USA). The number of positive labeled cells per square millimeter (cells/mm^2^) was manually counted by a blinded investigator. The Imaris software (Bitplane, Switzerland) was used to perform 3D reconstruction on the Z-series-scanning confocal images, and measure the volume of CD68^+^ or NLRP3^+^ punctures in microglia. The primary antibodies used for immunofluorescence included those against: rabbit anti-TGF-β1 (Abcam, USA), rat anti-MBP (Millipore, USA), goat anti-Iba1 (Wako, Japan), rabbit anti-Iba1 (Wako, Japan), rat anti-CD68 (Bio-Rad, USA), rabbit anti-GSDMD (Abcam, USA), rabbit anti-ASC (Cell Signaling Technology, USA), rabbit anti-IL-1β (Abcam, USA), rabbit anti-Smad3 (Cell Signaling Technology, USA), rabbit anti-NF-κB (Cell Signaling Technology, USA), rabbit anti-p-ERK1/2 (Cell Signaling Technology, USA), mouse anti-NLRP3 (AdipoGen, USA).

### Terminal transferase dUTP nick end labeling (TUNEL) staining

The cell apoptosis in brain slices and microglial climbing slices was assessed by TUNEL in situ cell death detection kit (Roche, Germany) according to the manufacturer’s instructions. Briefly, after permeabilized with 0.3% Triton-X100 and blocked with 10% bovine serum albumin, the slices were incubated with TUNEL reaction mixture for 1 h. Then, the cell climbing slices were mounted with an antifade mounting medium with DAPI, while the brain slices were further incubated with Iba1 primary antibody overnight at 4 °C and corresponding secondary antibody for 1 h before mounting. The images of stained cells were acquired with a laser scanning confocal microscope (Olympus FV500, Japan).

### Western blot

The samples of primary cultured microglia and isolated injury lesion (including 2 mm of surrounding tissue) from corpus callosum were lysed using RIPA buffer (Thermo Fisher Scientific, USA) supplemented with PMSF (Servicebio, China) and protease inhibitor cocktail (Thermo Fisher Scientific, USA). The protein concentration was determined using a BCA Kit (Beyotime, China). Samples containing 20–30 μg total proteins were loaded on 10–15% SDS‐PAGE gels. After electrophoresis, the proteins were transferred to the 0.22 or 0.45 μm nitrocellulose membrane (Millipore, USA). The nonspecific binding was blocked by 5% nonfat milk in Tris‐buffered saline containing 0.1% Tween‐20 (TBST) for 1 h at room temperature. The membrane was then incubated with corresponding primary antibodies (Additional file 3) overnight at 4 °C. After washed with TBST, membranes were incubated with secondary antibody (Jackson, USA) for 1 h at room temperature. Images were detected using an imaging system (Bio‐Rad, USA) with enhanced chemiluminescence kits (Advansta, USA). The bands were analyzed blindly using ImageJ software to obtain the optical density (OD) of signal. The value was expressed as the ratio of OD of the tested protein to OD of GAPDH (internal reference). The primary antibodies used for Western blot included those against: rabbit anti-GSDMD (Abcam, USA), mouse anti-Caspase-1-p20 (Adipogen, USA), rabbit anti-NLRP3 (Cell Signaling Technology, USA), rabbit anti-ASC (Cell Signaling Technology, USA), rabbit anti-IL-1β (ABclonal, China), mouse anti-Bax (Santa, USA), rabbit anti-Bcl-2 (Abcam, USA), rabbit anti-iNOS (ABclonal, China), goat anti-CD206 (R&D system, USA), rabbit anti-NF-κB (Cell Signaling Technology, USA), rabbit anti-p-NF-κB (Cell Signaling Technology, USA), rabbit anti-ERK1/2 (Cell Signaling Technology, USA), rabbit anti-p-ERK1/2 (Cell Signaling Technology, USA). All primary images of Western blots were presented in Additional file 4.

### Scanning electron microscopy

The primary cultured microglia on cover glass in different groups were fixed with 2.5% glutaraldehyde in 0.1 M PBS (Servicebio, China) for 2 h at room temperature, and then post-fixed with 1% OsO4 in 0.1 M PBS (pH 7.4) for 1–2 h. Samples were dehydrated through a graded ethanol series (30%, 50%, 70%, 80%, 90%, 95%, 100%), dried using critical point dryer, and sputter-coated with gold for 30 s. Images were captured using a scanning electron microscope (HITACHI Regulus 8100, Japan).

### Enzyme-linked immunosorbent assay (ELISA)

The concentration of IL-1β cytokine in conditioned medium of cultured microglia was measured with corresponding ELISA kit (Dakewe, China) according to manufacturer’s instructions. Briefly, diluted samples and cytokine standards were added to the coated wells of 96-well plates. Then, each well was incubated with diluted biotinylated antibody for 90 min and streptavidin-HRP for 30 min at 37 °C. After rinsed with washing buffer, the samples were added with TMB and Stop solution in order. The optical density of each well was measured by an enzyme-labeled instrument (Thermo Fisher Scientific, USA) at wavelength of 450 nm.

### Cytometric bead array (CBA)

The concentration of TNF-α and IL-6 cytokines in conditioned medium of cultured microglia was evaluated with the CBA Mouse Th1/Th2/Th17 Cytokine Kit (BD, USA) following manufacturer’s guidance. Briefly, diluted samples and cytokine standards were prepared and mixed with cytokine capture microspheres. Then, the mixture was incubated for 3 h at room temperature and washed with washing buffer before measurement. BD Accuri C6 flow cytometer was used to collect data, and the results were analyzed using FCAP Array V3 data analysis software.

### Lactate dehydrogenase (LDH) assay

The cell apoptosis of treated microglia was also measured by a commercial lactate dehydrogenase (LDH) kit according to manufacturer’s instructions (Beyotime, China). Briefly, supernatant of cultured microglia in different groups were collected and added to a 96-well plate, then, each well was incubated with 60 μL of LDH detection working solution for 30 min at room temperature. The optical density of each well was measured by an enzyme-labeled instrument (Thermo Fisher Scientific, USA) at wavelength of 490 nm.

### Statistical analysis

All of the data collected were processed randomly and appropriately blocked in this study. All of the data were expressed as the mean ± SEM except for LFB scoring analysis. LFB scoring was presented as the median with corresponding interquartile range. The differences among varied groups of mice in the escape latency were determined by two‐way ANOVA with repeated measures followed by Bonferroni post-test. The differences between varied groups in LFB staining were examined by Mann–Whitney *U* test. Other statistical differences among groups were measured by either the two-tailed Student’s t-test or one-way ANOVA with the Tukey post-test. Data analyses were performed using GraphPad Prism software version 8.0. A value of *p* < 0.05 was considered to be statistically significant.

## Results

### TGF-β1 accumulated in the injury focus of LPC-induced demyelinating model

**Fig. 1:**
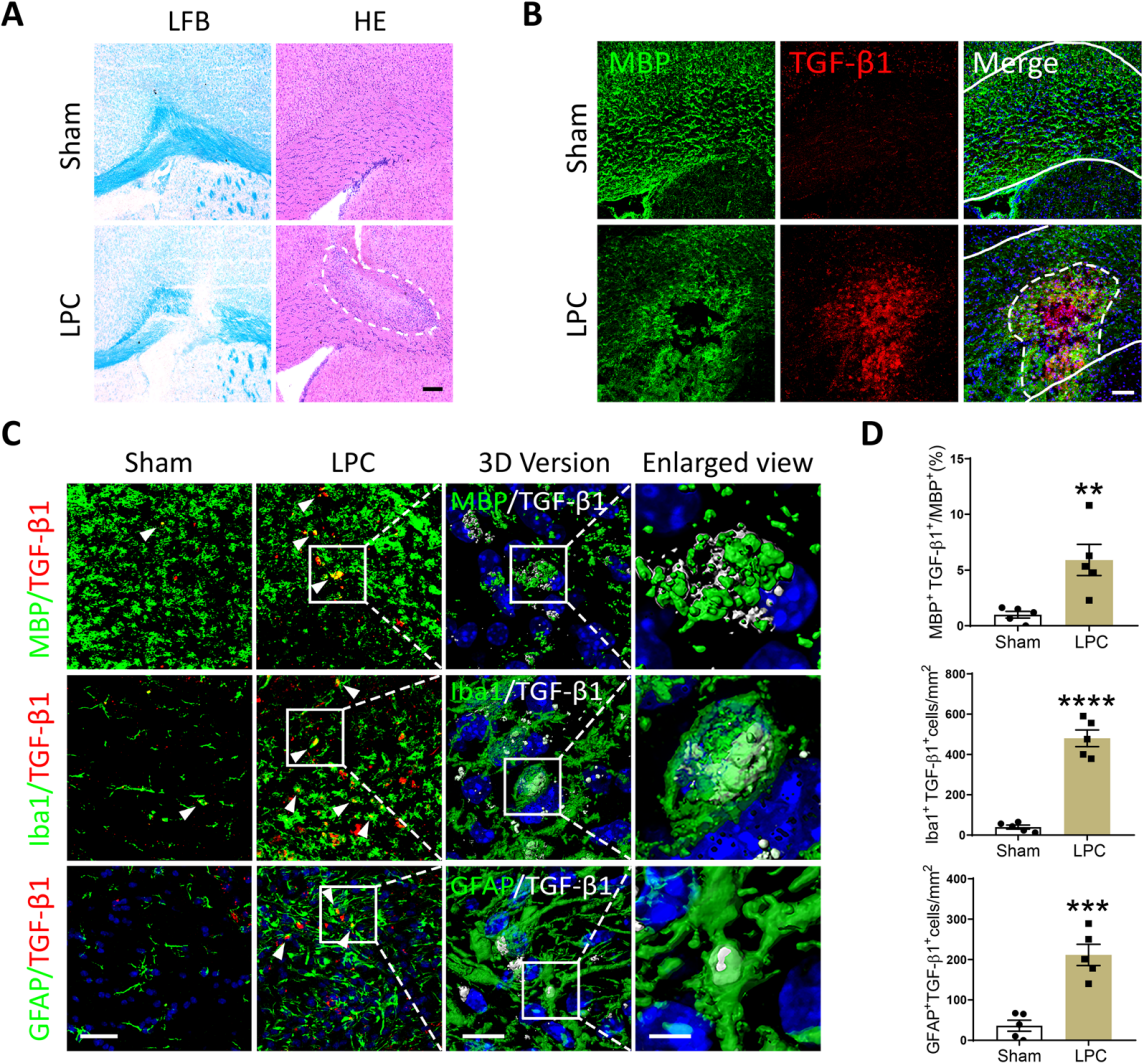
TGF-β1 accumulated in the demyelinating focus induced by LPC-injection. **A** Representative images of LFB and HE staining in the brain of LPC-modeling and Sham mice, scale bar = 200 μm. The lesion area was outlined with a white dotted line in HE staining. **B** Representative immunofluorescent staining of MBP and TGF-β1 in the corpus callosum of LPC-modeling and Sham mice, scale bar = 80 μm. The corpus callosum was outlined with white solid lines, while the lesion area was outlined with a white dotted line. **C** Representative confocal images of TGF-β1 with MBP, Iba1, and GFAP (scale bar = 30 μm), 3D reconstructed images (scale bar = 10 μm), and corresponding local enlarged views (scale bar = 3 μm) in the corpus callosum of LPC-modeling and Sham mice. White arrowheads pointed to the immunofluorescent double-labeling cells. **D** Quantitative analysis of MBP^+^TGF-β1^+^/MBP^+^, Iba1^+^TGF-β1^+^cells, and GFAP^+^TGF-β1^+^cells by immunofluorescence. Unpaired Student’s *t*-test was used for statistical analysis. **P < 0.01, ***P < 0.001, ****P < 0.0001 versus Sham group. *N* = 6 per group

We constructed a focal demyelinating mouse model by stereotaxically injecting concentrated LPC into the corpus callosum of brain as described previously [6]. The local demyelination was characterized by obvious disappearance of myelinated fibers, which was evaluated by LFB staining 10 days post modeling (Fig. 1A). Detected by HE staining, evident inflammatory cell infiltration was also involved in this demyelinating model (Fig. 1A). Interestingly, TGF-β1 was found accumulated in the demyelinated injury focus by double-immunostaining of MBP (a principal myelin marker) and TGF-β1. Comparatively, the expression of TGF-β1 maintained at a low level in Sham mice (Fig. 1B). To further confirm the specific cell types of expressing higher TGF-β1 after modeling, we double-labeled TGF-β1 with the myelin marker MBP, microglia marker Iba1, and astrocyte marker GFAP, respectively. As expected, significantly increased co-localizations of TGF-β1 with MBP, Iba1, and GFAP in the lesion were observed in LPC-modeling group versus Sham group (Fig. 1C, D). The z-stack 3D reconstructions of above images in LPC group were performed and demonstrated in Fig. 1C.

### Preventative administration of TGF-β1 alleviated the demyelinating injury and microglial activation in LPC-modeling mice.

**Fig. 2:**
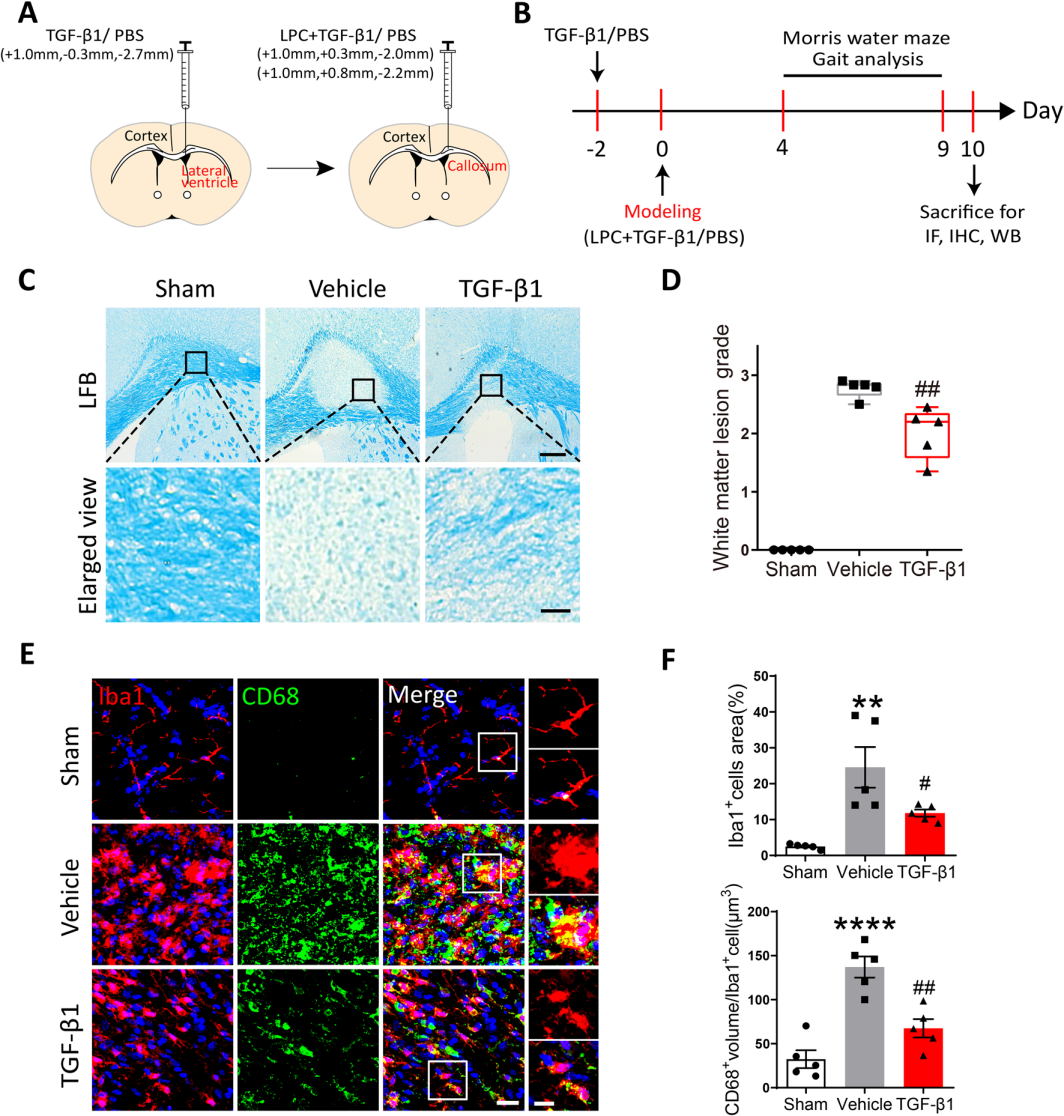
Preventative application of TGF-β1 ameliorated the severity of demyelination and microglial activation in LPC-modeling mice. **A** A schematic diagram showing the procedure and position of brain stereotactic injection. **B** The timeline of LPC-modeling, TGF-β1 application, behavioral tests, and tissues harvest. **C** Representative images of LFB staining in the corpus callosum among three groups, scale bar = 200 μm. Local enlarged views were presented, scale bar = 30 μm. **D** Quantitative analysis of LFB score was performed using Mann–Whitney *U* test. ##*P* < 0.01 versus Vehicle group. *N* = 5 per group. **E** Representative confocal images of Iba1 and CD68 in the corpus callosum among three groups, scale bar = 20 μm. Local enlarged views were presented, scale bar = 10 μm. **F** Quantitative analysis of the relative area of Iba1^+^cells and the volume of CD68^+^punctures in microglia, respectively. One-way ANOVA was used for statistical analysis followed by Tukey’s multiple comparisons test. **P < 0.01, ****P < 0.0001 versus Sham group; #*P* < 0.05, ##*P* < 0.01 versus Vehicle group. *N* = 5 per group

To examine whether preventative treatment of TGF-β1 played a protective role in LPC-induced lesion, we applied TGF-β1 two days before and on the day of LPC-modeling, respectively (Fig. 2A). The experimental design procedure in order of time was illustrated in Fig. 2B. We confirmed the disruption of myelin in the injected site by LFB staining at 10 days after LPC modeling. The lesion in TGF-β1 mice exhibited more marked vacuoles (grade 2) rather than the disappearance of myelinated fibers (grade 3) observed in Vehicle mice (Fig. 2C). The results of quantitative scoring demonstrated that TGF-β1 mitigated histological damage in corpus callosum to some extent, with significantly lower white matter lesion grade versus Vehicle group (Fig. 2D).

Microglial activation, characterized by hypertrophic morphology and enhanced release of inflammatory mediators, is known to impair myelination in different models of white matter injury [34–36]. Herein, we doubled-labeled microglia in brain slices with general microglia marker Iba1, and phagocytosis marker CD68 which was robustly expressed in activated microglia. At 10 days post modeling, the percent of Iba1^+^cells area to total lesion area and the volume of CD68^+^punctures in Iba1^+^cell were remarkably elevated in Vehicle group. Yet, TGF-β1-treated mice showed significantly less relative area of Iba1^+^cells and CD68^+^punctures volume in microglia compared with Vehicle mice, which indicated the decrease in microglial activation and its phagolysosome activity (Fig. 2E, F). However, the protein levels of pro-inflammatory marker iNOS and anti-inflammatory marker CD206 quantified by Western blot, were found consistent among three groups (Additional file 1: Fig. S1A, B). In brief, it was indicated that TGF-β1 attenuated neuroinflammation possibly through regulating microglial phagolysosome activity, while without switching microglia between pro-inflammatory and anti-inflammatory phenotype.

### Preventative use of TGF-β1 mitigated neuroinflammation in LPC-modeling mice via reducing the pyroptosis and apoptosis of microglia

**Fig. 3:**
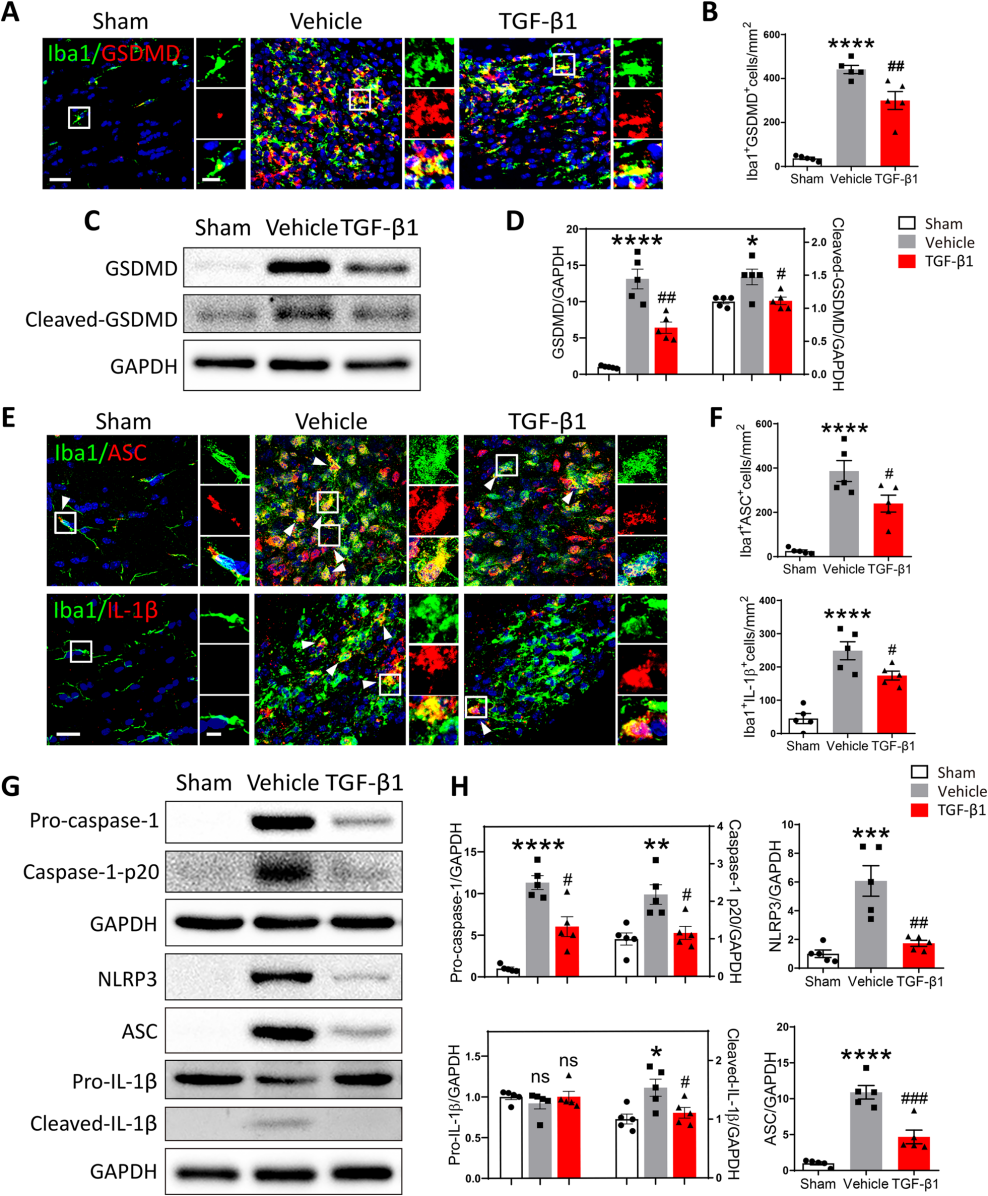
Preventative administration of TGF-β1 attenuated the pyroptosis of microglia in LPC-modeling mice. **A** Representative confocal images showing Iba1 and GSDMD in the corpus callosum among different groups, scale bar = 30 μm. Local enlarged views were presented, scale bar = 10 μm. **B** Quantitative analysis of the number of Iba1^+^GSDMD^+^cells was performed using One-way ANOVA followed by Tukey’s multiple comparisons test. ****P < 0.0001 versus Sham mice; ##*P* < 0.01 versus Vehicle mice. *N* = 5 per group. **C** The protein expression of GSDMD and Cleaved-GSDMD in the corpus callosum among three group was detected by Western blot. **D** Quantitative analysis of Western blot was performed using One-way ANOVA followed by Tukey’s multiple comparisons test. *P < 0.05, ****P < 0.0001 versus Sham group; #*P* < 0.05, ##*P* < 0.01 versus Vehicle group. *N* = 5 per group. **E **) Representative confocal images showing Iba1 with ASC or IL-1β in the corpus callosum among different groups, scale bar = 20 μm. Local enlarged views were presented, scale bar = 5 μm. White arrowhead pointed to the immunofluorescent double-labeling cells. **F** Quantitative analyses of the number of Iba1^+^ASC^+^cells and Iba1^+^IL-1β^+^cells were performed using One-way ANOVA followed by Tukey’s multiple comparisons test. ****P < 0.0001 versus Sham mice; #*P* < 0.05 versus Vehicle mice. *N* = 5 per group. **G** The protein expression of Pro-caspase-1, Caspase-1-p20, NLRP3, ASC, IL-1β, and Cleaved-IL-1β in the corpus callosum among three groups was detected by Western blot. **H** Quantitative analysis of Western blot was performed using One-way ANOVA followed by Tukey’s multiple comparisons test. *P < 0.05, **P < 0.01, ***P < 0.001, ****P < 0.0001, n.s. no significance versus Sham group; #*P* < 0.05, ##*P* < 0.01, ###*P* < 0.001, n.s. no significance versus Vehicle group. *N* = 5 per group

It was reported that microglia played a vital role in inflammatory processes through pyroptosis [18, 37]. Gasdermin D (GSDMD), a 487 amino acid cytoplasmic protein, has been discovered to form membrane pore upon caspase-1 activation and act as a key effector for pyroptosis [38]. To evaluate the pyroptosis process in microglia at 10 days post LPC injection, we double-labeled microglia by immunostaining for Iba1 and GSDMD. It was found that the number of Iba1^+^GSDMD^+^cells was significantly elevated in Vehicle mice, demonstrating LPC-induced demyelination was closely associated with microglial pyroptosis. Yet, treatment of TGF-β1 evidently downregulated the LPC-induced microglial pyroptosis characterized by fewer double-labeled cells (Fig. 3A, B). Consistent with above findings, protein levels of GSDMD and cleaved-GSDMD as determined by Western blot, were significantly decreased in TGF-β1-treated mice versus Vehicle mice (Fig. 3C, D).

NLRP3 inflammasome, consisting of NLRP3, apoptosis-associated speck-like protein (ASC) and Caspase-1, is a multiprotein complex that mediates the activation of caspase-1, which subsequently promotes Gasdermin D-mediated membrane pore formation, as well as the maturation and release of IL-1β and IL-18 [15]. In our study, it was observed that LPC-injection greatly upregulated the expression of ASC and IL-1β in Iba1^+^microglia, detected by double-labeling immunofluorescence. While the number of Iba1^+^ASC^+^ cells and Iba1^+^IL-1β^+^ cells were both downregulated in TGF-β1-treated mice compared to that in Vehicle mice (Fig. 3E, F). Furthermore, the protein levels of NLRP3, ASC, Pro-caspase-1, Caspase-1-p20, IL-1β and Cleaved-IL-1β among different groups were analyzed by Western blot. In line with the results of immunostaining, the NLRP3 inflammasome was evidently activated in Vehicle group, which was illustrated by the increased protein level of each component in NLRP3 inflammasome. Interestingly, as shown in Fig. 3G, H, preventative administration of TGF-β1 significantly alleviated the elevated expression of NLRP3, ASC, Pro-caspase-1, Caspase-1-p20 and Cleaved-IL-1β induced by LPC-injection to a certain extent.

Apart from anti-pyroptotic effect, the anti-apoptotic effect of TGF-β1 was also evaluated in the current research. Apoptosis is a form of programmed cell death, characterized by cell shrinkage and membrane blebbing [39]. Recent studies suggested that inflammasomes induced not only pyroptosis, but also apoptosis [40]. As shown in Additional file 1: Fig. S2A, C, LPC-induced demyelination was accompanied with pronounced apoptosis of microglia, which was examined by double-labeling of Iba1 and TUNEL, and corresponding z-stack 3D reconstruction. Yet, the number of Iba1^+^TUNEL^+^cells was found significantly reduced in TGF-β1-treated group compared to Vehicle group. Western blot further confirmed that, TGF-β1 attenuated the increased ratio of Bax (pro-apoptotic protein) to Bcl-2 (anti-apoptotic protein) induced by LPC-injection (Additional file 1: Fig. S2B, D).

Together, above data suggested that preventative use of TGF-β1 notably mitigated the pyroptosis and apoptosis of microglia, which could possibly attenuate the neuroinflammation in LPC-modeling mice.

### Preventative administration of TGF-β1 ameliorated cognitive dysfunction and motor deficit in LPC-modeling mice

**Fig. 4:**
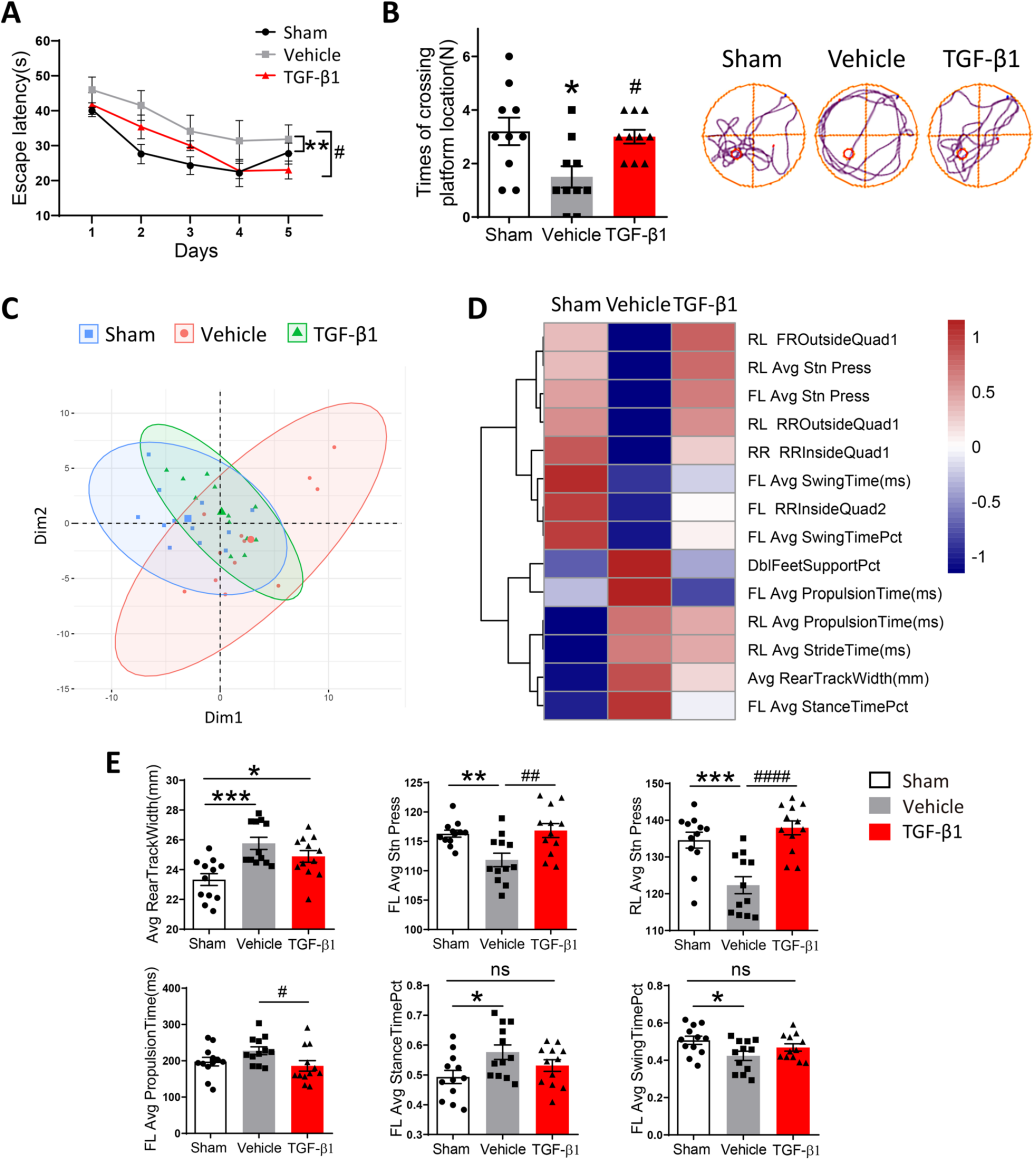
Preventative administration of TGF-β1 rescued the cognitive deficit and locomotion disorder in LPC-modeling mice. **A** The escape latency of Sham mice and LPC-modeling mice treated with PBS or TGF-β1 during acquisition phase of Morris water maze test. Two‐way ANOVA with repeated measures was used for statistical analysis followed by Bonferroni posttests. **P < 0. 01 versus Sham mice; #*P* < 0.05 versus Vehicle mice. *N* = 10 per group. **B** The times of crossing the platform location in probe trial of Morris water maze test. Representative movement trails of three groups were shown. One-way ANOVA was used for statistical analysis followed by Tukey’s multiple comparisons test. *P < 0.05 versus Sham mice; #*P* < 0.05 versus Vehicle mice. *N* = 10 per group. **C**The Principal Component Analysis (PCA) of motor-associated indexes in Sham mice and LPC-modeling mice treated with PBS or TGF-β1 in TreadScan test. **D** The heatmap involved some gait parameters with significant difference among three groups in TreadScan test. **E** The specific statistical charts of Average rear track width, FL average propulsion time, FL average stance time percent, FL average stance press, FL average swing time percent and RL average stance press among three groups. One-way ANOVA was used for statistical analysis followed by Tukey’s multiple comparisons test. *P < 0.05, **P < 0.01, ***P < 0.001, n.s. no significance versus Sham mice; #*P* < 0.05, ##*P* < 0.01, ####*P* < 0.0001 versus Vehicle mice. *N* = 12 per group

To assess the effects of preventative TGF-β1 treatment on cognition in LPC-modeling mice, we conducted Morris water maze test to evaluate spatial learning and memory capabilities in mice among different groups. During acquisition episode, Vehicle group exhibited poorer escape abilities versus Sham group, while TGF-β1 treatment significantly ameliorated LPC-induced learning dysfunction (Fig. 4A). In probe test with the platform removed, the times of crossing platform position in Vehicle mice were evidently fewer than Sham and TGF-β1-treated mice (Fig. 4B). Above results indicated that the spatial learning and memory retrieval capabilities in LPC-modeling mice were impaired, while TGF-β1 treatment improved LPC-induced cognitive dysfunction.

Moreover, the locomotion performance in modeling mice was evaluated by unbiased TreadScan. The results of Principal Component Analysis (PCA), a mathematical algorithm that reduced the dimensionality of data while retained most of the variation in data set [41], showed TGF-β1-treated group was different from Vehicle group but similar to Sham group in PC1 (Fig. 2C). Yet, as behavioral tests did have a lot of variability among animals, it was difficult to clearly distinguish different groups in our PCA analysis. Many parameters measuring gaits and mobility were classified and demonstrated in the form of cluster heatmap (Fig. 2D). Specifically, TGF-β1-treated mice exhibited narrower Average rear track width, shorter FL average propulsion time, smaller FL average stance time percent, greater FL average stance press, FL average swing time percent, and RL average stance press, with statistical difference or trend as compared to Vehicle mice (Fig. 2E). Overall, above analyses suggested that preventative application of TGF-β1 could partially improve the locomotion performance in LPC-modeling mice.

### Therapeutic use of TGF-β1 alleviated the pyroptosis of microglia and cognitive dysfunction in LPC-modeling mice

**Fig. 5:**
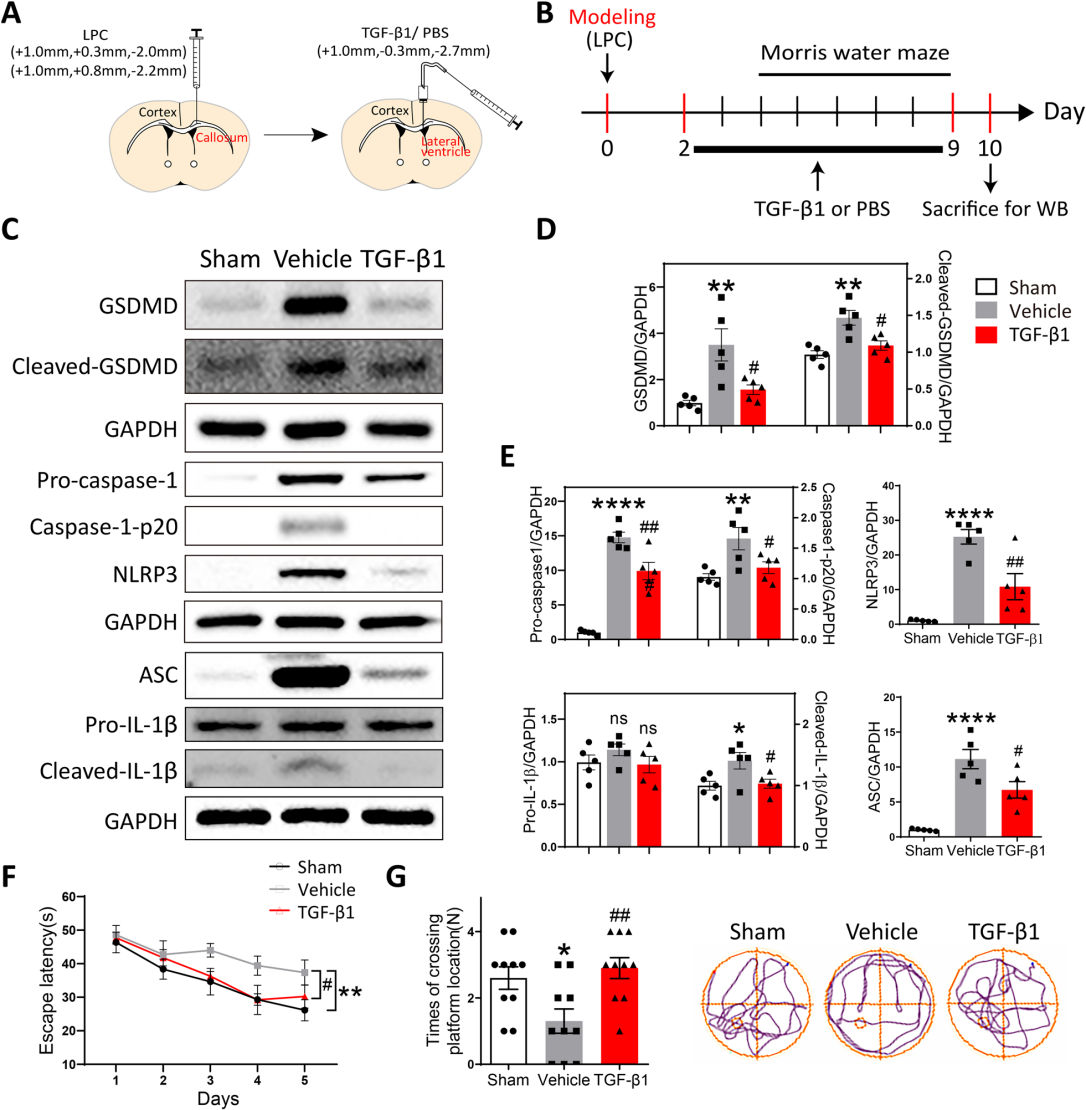
Therapeutic use of TGF-β1 alleviated the pyroptosis of microglia LPC-modeling mice. **A** A schematic diagram showing the procedure and position of brain stereotactic injection. **B** The timeline of LPC-modeling, TGF-β1 application, behavioral tests, and tissues harvest. **C** The protein level of GSDMD, Cleaved-GSDMD, Pro-caspase-1, Caspase-1-p20, NLRP3, ASC, Pro-IL-1β, and Cleaved- IL-1β in the corpus callosum among three group was examined by Western blot. **D**, **E** Quantitative analysis of Western blot was performed using One-way ANOVA followed by Tukey’s multiple comparisons test. **P < 0.01, ****P < 0.0001, n.s. no significance versus Sham group; #*P* < 0.05, ##*P* < 0.01, n.s. no significance versus Vehicle group. *N* = 5 per group. **F** The escape latency among three groups of mice during acquisition phase of Morris water maze test. Two‐way ANOVA with repeated measures was used for statistical analysis followed by Bonferroni posttests. **P < 0. 01 versus Sham mice; #*P* < 0.05 versus Vehicle mice. *N* = 10 per group. **G** The times of crossing the platform location in probe trial of Morris water maze test. Representative movement trails of three groups were shown. One-way ANOVA was used for statistical analysis followed by Tukey’s multiple comparisons test. *P < 0.05 versus Sham mice; ##*P* < 0.01 versus Vehicle mice. *N* = 10 per group

To examine whether therapeutic administration of TGF-β1 also played a protective role in LPC-induced lesion, we delivered TGF-β1 to the lateral ventricle of modeling-mice for a week using a microdose cannula (Fig. 5A). The experimental design procedure in order of time was illustrated in Fig. 5B. The application of TGF-β1 post LPC-injection partially ameliorated the process of activated pyroptosis, as identified by the decreased protein levels of pyroptosis-associated markers (GSDMD, cleaved-GSDMD, Pro-caspase-1, Caspase-1-p20, NLRP3, ASC, and cleaved-IL-1β) in TGF-β1-treated group versus Vehicle group (Fig. 5C–E). Consistently, using immunostaining and Western blot, we also detected relieved apoptosis of microglia in TGF-β1-treated mice versus Vehicle mice, characterized by reduced number of Iba1^+^TUNEL^+^cells and downregulated ratio of Bax to Bcl-2 (Additional file 1: Fig. S3). Furthermore, the results of Morris water maze indicated that therapeutic treatment of TGF-β1 significantly improved the escape abilities in LPC-modeling mice as compared to Vehicle mice, especially in the last three days during acquisition test (Fig. 5F). This finding could be explained that it took time for TGF-β1 applied post LPC-modeling to exert its protective functions on cognitive disorders. As well, TGF-β1-treated mice presented more times of crossing platform position versus Vehicle mice during probe test (Fig. 5G). In summary, it was found that not only preventative but also therapeutic administration of TGF-β1 could suppress the inflammatory pyroptosis of microglia, and ameliorate the cognitive dysfunction in LPC-modeling mice.

### TGF-β1 suppressed LPS-induced pyroptosis in primary cultured microglia

**Fig. 6:**
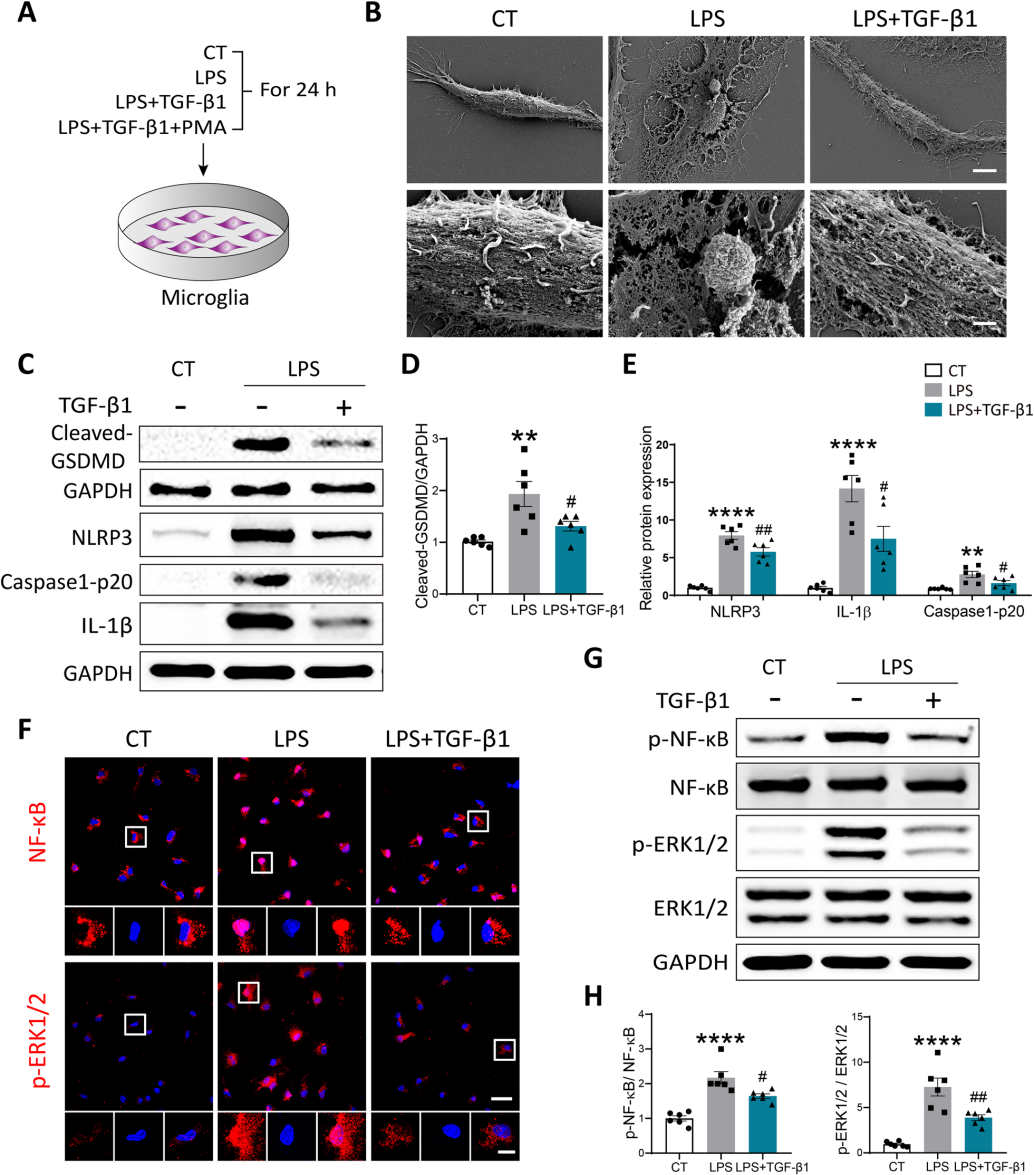
TGF-β1 relieved the pyroptosis of primary cultured microglia induced by LPS in vitro. **A** A schematic diagram demonstrating different groups of primary cultured microglia. **B** Representative scanning electron microscopy (SEM) images showing morphological changes of cultured microglia under different treatments, scale bar = 5 μm. Higher magnification images were presented, scale bar = 1 μm. **C** The protein expression of pyroptosis-associated markers (Cleaved-GSDMD, NLRP3, Caspase-1-p20, and IL-1β) in cultured microglia was detected by Western blot. **D**, **E** Quantitative analysis of Western blot was conducted using One-way ANOVA followed by Tukey’s multiple comparisons test. **P < 0.01, ****P < 0.0001 versus CT group; #*P* < 0.05, ##*P* < 0.01 versus LPS group. *N* = 6 per group. **F** Representative confocal images showing NF-κB and p-ERK1/2 in cultured microglia, scale bar = 30 μm. Local enlarged views were demonstrated, scale bar = 10 μm. **G** The protein expression of p-NF-κB, NF-κB, p-ERK1/2, and ERK1/2 in cultured microglia among three group was examined by Western blot. **H** Quantitative analysis of Western blot was performed using One-way ANOVA followed by Tukey’s multiple comparisons test. ****P < 0.0001 versus CT group; #*P* < 0.05, ##*P* < 0.01 versus LPS group. *N* = 6 per group

To directly address whether TGF-β1 exerted its protective functions on LPC-induced lesion through suppressing the pyroptosis of microglia, we employed lipopolysaccharide (LPS) in primary cultured microglia to induce activation of NLRP3 inflammasome in vitro as previously described [15] (Fig. 6A)*.* Activation of canonical TGF-β signaling cascade is typically initiated by c-terminal phosphorylation of SMAD2 and SMAD3, as well as complex formation with SMAD4. The activated SMAD complexes translocate into the nucleus to regulate the transcription of multiple target genes in cooperation with co-activators and co-repressors [42–44]. The immunostaining images showed that Smad3 was evidently translocated into the nuclei of cultured microglia within 1 h after addition of TGF-β1 (Additional file 1: Fig. S4A). The morphological changes of pyroptosis are characterized by cellular swelling, emergence of bubbles from the plasma membrane and cell membrane rupture [45, 46]. Consistent with the published studies [45–47], using scanning electron microscopy, we observed emergence of typical bubbles from plasma membrane and obvious cell membrane rupture in microglia treated with LPS for 24 h. Yet, TGF-β1 administration greatly relieved above pyroptosis-characteristic morphological changes caused by LPS (Fig. 6B). To further determine whether the pyroptosis of microglia was repressed by TGF-β1, we compared the protein expressions of Cleaved-GSDMD, NLRP3 inflammasome, and IL-1β among Control, LPS, and LPS + TGF-β1-treated groups. As shown in Fig. 6C–E, application of TGF-β1 notably attenuated the increased protein levels of Cleaved-GSDMD, NLRP3, Caspase-1-p20 and IL-1β in cultured microglia stimulated with LPS. In addition, TGF-β1 could also inhibit activated apoptotic process in cultured microglia exposed to LPS, which was identified by significantly decreased protein level of Bax and ratio of Bax to Bcl-2 (Additional file 1: Fig. S4B, C).

### TGF-β1 regulated LPS-induced pyroptosis in primary cultured microglia through ERK1/2 and NF-κB pathways

It was reported that NF-κB elevated GSDMD transcription by binding to two proximal sites in upstream of GSDMD promoter region [48]. Blockade of ERK1/2 and NF-κB activity, respectively, could largely attenuated monocarboxylate transporter 4 (MCT4)-induced pyroptosis [49]. In our study, to further investigate the specific molecular mechanism involved in the anti-pyroptotic effect of TGF-β1, we assessed the expression levels of NF-κB and ERK1/2 pathways markers among different groups by immunofluorescence and Western blot. As expected, LPS stimulation caused obvious translocation of NF-κB into the nuclei, and strikingly elevated fluorescence intensity of p-ERK1/2 in the nuclei. While treatment of TGF-β1 evidently reversed above changes in LPS-stimulated microglia (Fig. 6F). Consistently, the results of Western blot confirmed that TGF-β1 could significantly suppress the up-regulated ratio of p-NF-κB to NF-κB, and p-ERK1/2 to ERK1/2 in cultured microglia exposed to LPS (Fig. 6G, H).

**Fig. 7:**
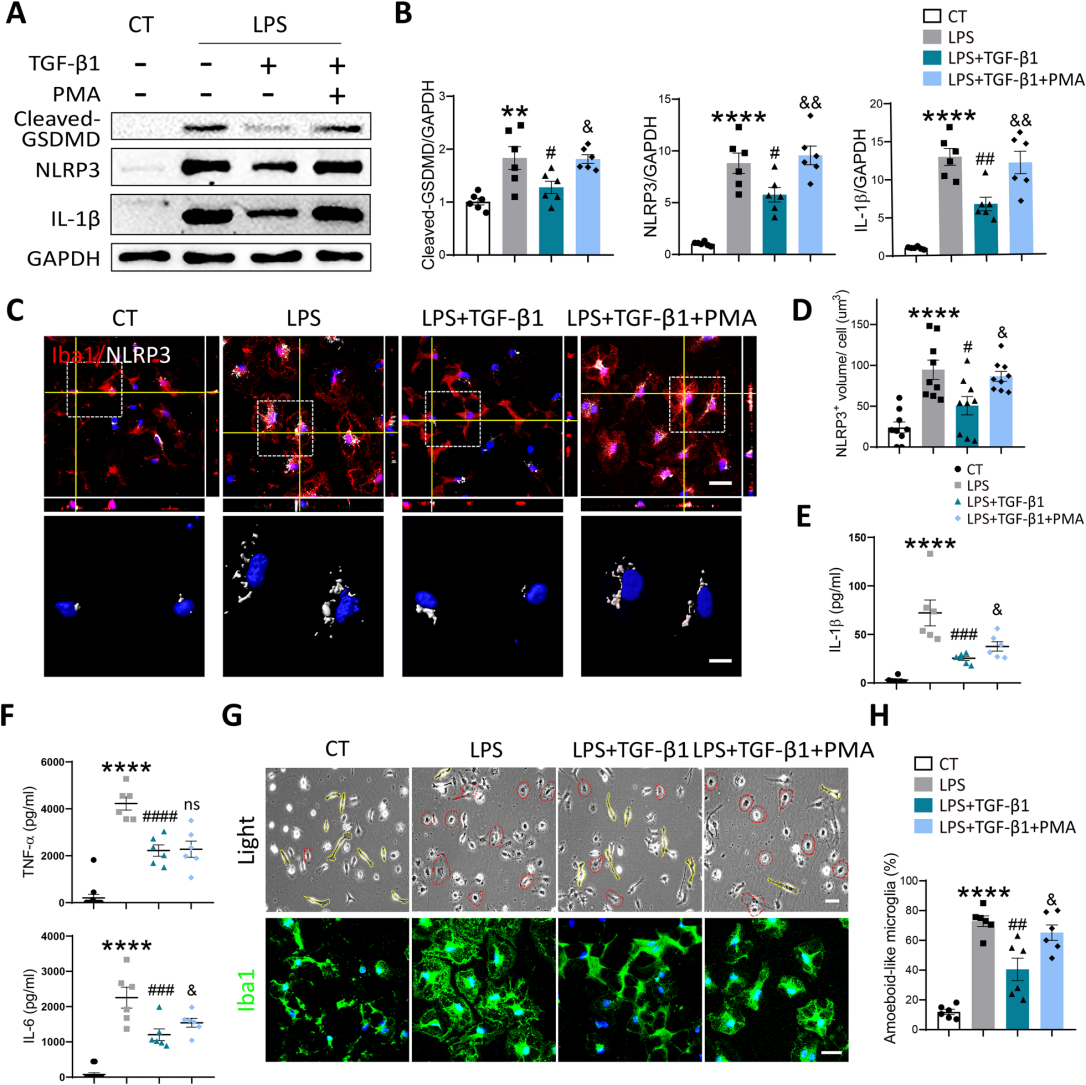
Activation of ERK1/2 and NF-κB pathways by PMA partially reversed the anti-pyroptotic effect of TGF-β1 in vitro. **A** The protein expression of Cleaved-GSDMD, NLRP3, and IL-1β in cultured microglia under different treatments was evaluated by Western blot. **B** Quantitative analysis of Western blot was performed using One-way ANOVA followed by Tukey’s multiple comparisons test. **P < 0.01, ****P < 0.0001 versus CT group; #*P* < 0.05, ##*P* < 0.01 versus LPS group; &*P* < 0.05, &&*P* < 0.01 versus LPS + TGF-β1 group. *N* = 6 per group. **C** Representative confocal images (scale bar = 30 μm) and 3D reconstructed images in high power field (scale bar = 10 μm) of Iba1 and NLRP3 in cultured microglia under different treatments. **D** Quantitative analysis of the volume of NLRP3^+^ punctures per cell in immunofluorescent staining. One-way ANOVA followed by Tukey’s multiple comparisons test was used for statistical analysis. ****P < 0.0001 versus CT group; # *P* < 0.05 versus LPS group, &*P* < 0.05 versus LPS + TGF-β1 group. *N* = 9 per group. **E** The level of IL-1β in microglial CM under different treatments was analyzed by ELISA. Quantitative analysis was performed using One-way ANOVA followed by Tukey’s multiple comparisons test. ****P < 0.0001 versus CT group; ###*P* < 0.001 versus LPS group; &*P* < 0.05 versus LPS + TGF-β1 group. *N* = 6 per group. **F** The level of TNF-α and IL-6 in microglia-CM exposed to varied stimulations was measured by CBA. Quantitative analysis was performed using One-way ANOVA followed by Tukey’s multiple comparisons test. ****P < 0.0001 versus CT group; ###*P* < 0.001, ####*P* < 0.0001 versus LPS group; &*P* < 0.05, n.s. no significance versus LPS + TGF-β1 group. *N* = 6 per group. **G** Representative images of bright field (scale bar = 40 μm) and immunostaining with Iba1 (scale bar = 30 μm) in cultured microglia exposed to varied treatments. **H** Quantitative analysis of the percent of amoeboid-like microglia in bright-field images. ****P < 0.0001 versus CT group; ##*P* < 0.01 versus LPS group; &*P* < 0.05 versus LPS + TGF-β1 group. *N* = 6 per group

As shown in Fig. 6A, to further explore the involvement of ERK1/2 and NF-κB pathways in anti-pyroptotic function of TGF-β1, we additionally applied phorbol 12‑myristate 13‑acetate (PMA) in our experiment. PMA is a strong agonist both for ERK1/2 and NF-κB pathway [28], which was verified by the results of immunofluorescence and Western blot in present study (Additional file 1: Fig. S5A-C). As expected, the downregulated protein levels of Cleaved-GSDMD, NLRP3, and IL-1β in cultured microglia exposed to LPS + TGF-β1, were largely abolished by addition of PMA (Fig. 7A, B). By performing immunostaining of NLRP3 among different groups, we found that TGF-β1 treatment significantly reduced the average volume of NLRP3^+^punctures in cultured microglia exposed to LPS. However, activation of ERK1/2 and NF-κB pathway by PMA reversed this effect, as shown in the confocal scanning and z-stack 3D reconstructed images (Fig. 7C, D). To further assess the anti-inflammatory function of TGF-β1, we conducted ELISA and Cytometric Bead Array (CBA) test to measure the level of IL-1β, TNF-α, and IL-6 in microglial conditioned medium (CM) among different groups. As expected, TGF-β1 markedly decreased the level of above three pro-inflammatory cytokines in the CM of microglia exposed to LPS. Extra addition of PMA partially reversed these trends in the expression of IL-1β and IL-6, while without influence on TNF-α (Fig. 7E, F).

The participation of ERK1/2 and NF-κB pathway in the anti-apoptotic effect of TGF-β1 was also evaluated by TUNEL staining. The results presented that TGF-β1 treatment significantly reduced the elevated number of TUNEL^+^cells in cultured microglia stimulated with LPS. Yet, the administration of PMA reversed this change to a certain extent (Additional file 1: Fig. S5E). Similarly, additional activation of ERK1/2 and NF-κB pathway increased the LDH release from treated microglia, which represented enhanced apoptotic process (Additional file 1: Fig. S5D). As the cell morphology of microglia was accessible to external stimulation, we subsequently observed the morphological transformation of cultured microglia among different groups by optical microscope and immunofluorescence staining of Iba1. In normal condition, control microglia possessed ramified cell morphology with fine processes, while after exposure to LPS, most of cells presented a rounded amoeboid-like appearance. TGF-β1 treatment evidently alleviated this amoeboid-like switch in LPS-stimulated microglia, which could be further abolished by PMA (Fig. 7G). The quantitative analysis on percent of amoeboid-like microglia in optical microscope images were performed and presented in Fig. 7H.

Collectively, above results verified our primary speculations that TGF-β1 could directly inhibit the pyroptosis process of microglia to reduce the release of proinflammatory cytokines and neuroinflammation via suppressing ERK1/2 and NF-κB pathways.

## Discussion

**Fig. 8:**
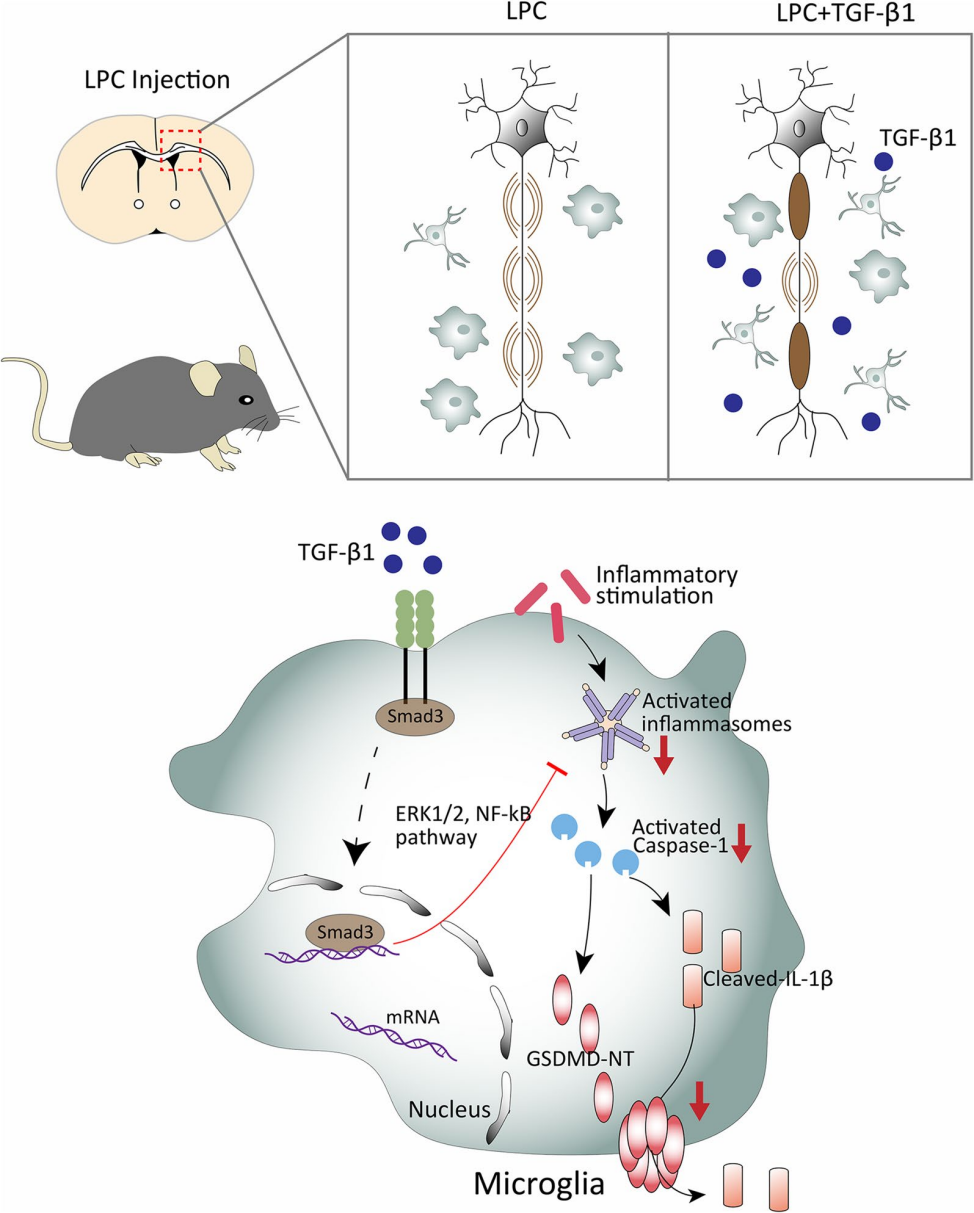
A schematic illustration showing the protective effects of TGF-β1 on LPC-induced demyelination and its underlying mechanisms. Brain stereotactic injection of LPC leads to evident demyelination and activated microglial pyroptosis in the corpus callosum of mice. Mechanistically, the external inflammatory stimulation activates the NLRP3 inflammasomes, which could cleave pro-caspase-1 to capspase-1-p20. Cleaved caspase-1 promotes the oligomerization of GSDMD to form membrane pores, as well as the maturation and release of IL-1β to extracellular space through membrane pores. Supplementary TGF-β1 binds to its receptor TGF-βRI/II and improves the translocation of its downstream molecule Smad3 into nuclei to exert subsequent functions. The upregulation of TGF-β1 signal pathway suppresses the process of pyroptosis in microglia by reducing the activated inflammasomes, cleaved-GSDMD, and IL-1β through ERK1/2 and NF-κB pathways. In brief, TGF-β1 could alleviate the neuroinflammation by reducing pro-inflammatory pyropsis of microglia and ultimately protect against demyelinating injury and cognitive disorder in LPC-induced demyelinating model

The important roles of TGF-β1 have been examined and reported in multiple CNS disorders, such as Alzheimer’s disease (AD) [50], stroke [51], motor neuron diseases [52], multiple sclerosis [53], etc. However, the underlying mechanisms of TGF-β1’s protective effects on demyelinating disease remain largely unknown. The present study revealed the influences of TGF-β1 administration on LPC-induced demyelinating lesion, and identified its effects on pyroptosis of microglia and potential molecular mechanisms. Our key findings included: (1) LPC-induced demyelination was accompanied with accumulation of TGF-β1 in the injury focus. (2) Endogenous supplement of TGF-β1 attenuated myelin damage and neuroinflammation via repressing the overactive proinflammatory pyroptosis of microglia. (3) Administration of TGF-β1 exhibited protective activities against cognitive disorder and motor deficit in LPC-modeling mice. (4) TGF-β1 repressed the overactive pyroptosis of LPS stimulated-microglia through regulating ERK1/2 and NF-κB signal pathways. To the best of our knowledge, this study is the first to indicate beneficial effects of TGF-β1 on LPC-induced demyelinating injury and its underlining molecular mechanisms. The schematic diagram of above findings was demonstrated in Fig. 8.

In CNS, under some conditions, TGF-β1 could protect neurons against damage induced by excitotoxins, hypoxia/ischemia, deprivation of trophic factors, and aggregates of amyloid beta (Aβ) [21]. It was also reported that circulating TGF-β1 had a protective role in the CNS and specifically promoted remyelination [54]. Instead, under some other conditions, accumulated or excessive TGF-β1 exhibited Alzheimer’s disease (AD)-like neuroinflammation and cerebrovascular dysfunction [55]. Contrary opinions were also proposed by another published study, that TGF-β1 was downregulated in 3 × Tg-AD mice, and administration of TGF-β1 could restore hippocampal synaptic plasticity and memory in AD model via the PI3K/Akt/Wnt/β-Catenin signaling pathway [50]. Actually, TGF-β1 may serve as a bidirectional cytokine in multiple neurology disorders, which is closely associated with its concentration, distribution, etc. It was worthy to note that in present study, the TGF-β1 was found intrinsically increased in the demyelinating lesion by immunostaining, which indicated its possible protective roles for LPC-induced injury. In many pathological conditions, some endogenous molecules could be upregulated and participate in protecting against the progression of pathology [56–58]. Yet, obviously, the intrinsic increase of TGF-β1 was not enough and compensatory to the inflammatory demyelination caused by LPC, probably due to its insufficient concentration and inappropriate distribution. Therefore, we determined to apply exogenous TGF-β1 to the modeling mice to further investigate potential protective functions of TGF-β1 on demyelinating lesion.

Pyroptosis is a proinflammatory form of programmed cell death that relies on the activity of cytosolic GSDMD driven by inflammasomes [59]. Upon activation, GSDMD.

transfers to plasma membrane and binds to inner membrane lipids, oligomerizing to form membrane pores, resulting in local cell swelling, membrane rupture, and extravasation of cytoplasmic damage-associated molecular patterns (DAMPs) [38, 60, 61]. Released DAMPs will further recruit immune cells and aggravate inflammatory cascade [62]. Meanwhile, some inflammatory cytokines, such as IL-1β and IL-18, could by cleaved by activated caspase-1 (the component of inflammasomes) and released to extracellular environment through GSDMD-formed membrane pores to play a proinflammatory role [17]. Pyroptosis is increasingly found to play important roles in the pathological changes of demyelinating diseases. It was reported that multiple inflammasome-associated proteins, including caspase-1, ASC, NLRP3, IL-18, and IL-1β, were notably expressed in MS lesions [63, 64]. Besides, IL-1β itself was able to increase the permeability of blood–brain barrier, facilitate leukocyte infiltration, and promote neurotoxicity in the EAE model [63]. Moreover, in EAE, deletion of inflammasome genes (e.g., ASC^−/−^ and caspase-1^−/−^) reduced the severity of disease, while NLRP3^−/−^ mice displayed variable outcomes [65, 66]. Treatment with VX765 (a caspase-1 inhibitor) could significantly improve neurobehavioral performance, reduce neuropathological severity, and diminish molecular indicators of inflammation in the EAE model [67]. For the first time, our study demonstrated that TGF-β1 administration could evidently alleviate demyelinating lesion and cognitive dysfunction in LPC-modeling mice, through suppressing the activated pyroptosis of microglia and neuroinflammation.

Inflammasome activation and pyroptosis have been reported existing in multiple cell types in CNS [68]. All of human microglia, neurons, and astrocytes could display robust NRLP3 inflammasome-associated responses [68]. Among them, microglia, vital mediators of innate immune responses following CNS injury [69], are considered to be the main cells where pyroptosis occurs in CNS [68, 70]. Compared to other cell types, microglia express higher levels of pattern recognition receptors (PRRs) which could recognize pathogen-associated molecular patterns (PAMPs) and DAMPs, and initiate pyroptosis cascade [68, 70]. Although it was increasingly reported that microglial pyroptosis participated in a variety of neuroinflammation-related diseases, the specific molecular and regulating mechanism of its occurrence have not been well understood. In the present study, we revealed that TGF-β1 could greatly ameliorate the LPS-stimulated pyroptosis of microglia, with decreased expressions of the inflammasome components NLRP3 and Caspase-1-p20, as well as proinflammatory cytokine IL-1β. Furthermore, it was verified that the anti-pyroptotic effect of TGF-β1 on microglia was mediated by the repression of ERK1/2 and NF-κB signal pathways.

Cell death is generally classified to three forms as apoptosis, pyroptosis, and necrosis. Among them, apoptosis is widely recognized as a kind of programmed cell death eliciting no inflammatory responses [71]. However, it was recently reported that in the absence of GSDMD, activated caspase‐1 induce apoptosis rather than pyroptosis [72, 73]. In GSDMD‐deficient macrophages, inflammasome formation led to rapid apoptotic morphological changes and activation of caspase‐3. The chemical dimerization of caspase‐1 induced pyroptosis in GSDMD‐sufficient cells, while induced apoptosis in GSDMD‐null/low cells [73]. In macrophages, the apoptosis‐induced activation of caspase‐1 and ‐8 led to GSDMD maturation, rendering apoptotic macrophages lytic, thereby could enhance IL‐1β release [74]. Consequently, we further evaluated the anti-apoptotic function of TGF-β1 in the present research. As expected, it was found that TGF-β1 could also relieve the activated apoptotic process of microglia in LPC-induced demyelinating model, possibly through suppressing the ERK1/2 and NF-κB signal pathways.

Apart from detrimental effects of microglial response in diseases, it is also known that microglia display a protective sensory mechanism to detect neural tissue damage in the form of neurodegeneration-associated molecular patterns (NAMPs), especially at the onset of neurodegeneration [75, 76]. In amyotrophic lateral sclerosis (ALS), available evidences suggest the coexistence of quite different roles for microglia, characterized by neuroprotective functions at early stages, and neurotoxic actions during disease progression [76]. Targeting the microglia has been the focus of neuroprotective strategies, which aims at modulating microglial reactivity in the attempt to improve the outcomes of animal disease models [77]. Therefore, in our LPC-induced demyelinating model, the time window to apply TGF-β1 targeting on microglia would also greatly influence the results observed. It is possible that applying TGF-β1 treatment in earlier time point might exert larger protective functions again neuroinflammation and demyelinating lesion in LPC model. Further and deeper studies would be performed to investigate and identify above speculations in the future. Moreover, it was worthy to note that in our study, microglia were robustly activated and proliferated when exposed to LPC stimulation, with evident increase in the relative area of Iba1^+^cells. At the same time, a portion of activated microglia were undergoing pyroptosis as well, which could lead to programmed cell death. In LPC-modeling, significantly increased number of microglia was ultimately present and observed by immunostaining, as microglia still maintained cell viability, expressed specific marker protein during early phase of pyroptosis, and possessed higher proportion of proliferation versus pyroptosis.

Despite the novelty of our findings that anti-pyroptotic effects of TGF-β1 attenuate the severity of demyelinating injury, there still remains several limitations in the current study. Firstly, as a model of MS, although the classic LPC-model well achieves the process of demyelination and activation of neuroinflammation, the demyelination region induced by LPC is limited to the injecting site, and its pathogenesis is not similar to any type of demyelinating disease [6]. Since the etiology and histopathology of MS is complex, other kinds of MS disease models including EAE and cuprizone diet all have parallel limitations. Secondly, we cannot exclude the possibility that TGF-β1 could exert its protective functions on demyelinating lesion through directly affecting oligodendrocytes, apart from through suppressing inflammatory pyroptosis of microglia. Further explorations on this hypothesis need to be conducted in the future.

## Conclusions

In summary, our study reveals that exogenous supplement of TGF-β1 protects against LPC-induced cognitive deficit and demyelinating lesion in modeling mice, through suppressing proinflammatory microglial pyroposis via NF-κB/ERK1/2 signal pathways.

Clinically, increasing or modulating the activity of TGF-β1 pathway may serve as a feasible and promising therapeutic strategy to demyelinating diseases.

## Supplementary Information

**Additional file 1.** Supplementary Figures S1–S5.

**Additional file 2.** R codes for Principal Component Analysis (PCA) and heatmap in TreadScan test.

**Additional file 3.** Information about antibodies applied in IF and WB.

**Additional file 4.** Raw images of Western blot.

## Data Availability

The datasets used and/or analysed during the current study are available from the corresponding author on reasonable request.

## Abbreviations

AD: Alzheimer’s disease
ADEM: Acute disseminated encephalomyelitis
ASC: Apoptosis-associated speck-like protein
Aβ: Amyloid beta
CBA: Cytometric bead array
CM: Conditioned medium
CNS: Central nervous system
DAMPs: Damage-associated molecular patterns
EAE: Experimental autoimmune encephalomyelitis
ELISA: Enzyme-linked immunosorbent assay
GSDMD: Gasdermin D
HE: Hematoxylin–eosin
HI: Hypoxic-ischemic
IF: Immunofluorescence
LDH: Lactate dehydrogenase
LFB: Luxol fast blue
LPC: Lysophosphatidylcholine
LPS: Lipopolysaccharide
MS: Multiple sclerosis
NMO: Neuromyelitis optica
PAMPs: Pathogen-associated molecular patterns
PCA: Principal component analysis
PFA: Paraformaldehyde
PRRs: Pattern recognition receptors
TGF-βs: Transforming growth factor betas
TUNEL: Terminal transferase dUTP nick end labeling
