# Supplementary material for: Transforming growth factor-β1 protects against LPC-induced cognitive deficit by attenuating pyroptosis of microglia via NF-κB/ERK1/2 pathways
Source: J Neuroinflammation. 2022 Jul 28;19:194. doi: 10.1186/s12974-022-02557-0 (PMC9336072; doi:10.1186/s12974-022-02557-0)
Supplement: Supplementary file 1 — Additional file 1. Supplementary Figures S1–S5. [file 12974_2022_2557_MOESM1_ESM.docx]

**Additional file 1**

**Supplementary Figures S1-S5**

**
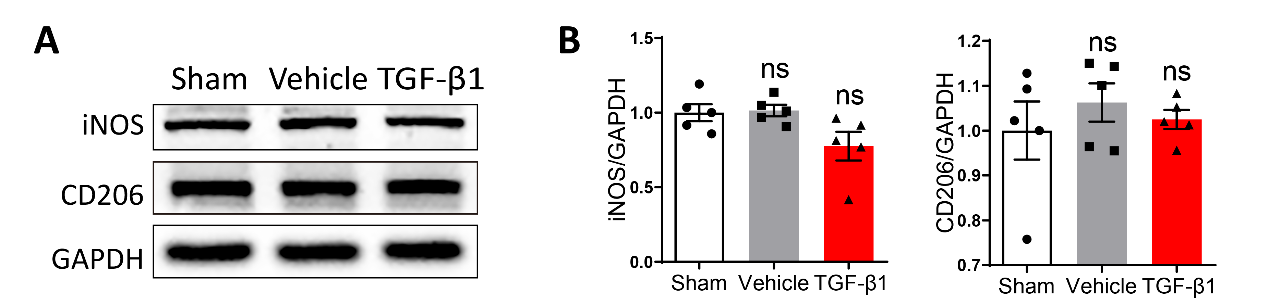
**

**Fig. S1 Preventative use of TGF-β1 did not influence the phenotype of microglia in LPC-modeling mice.**

(**A**) The protein level of iNOS and CD206 in the corpus callosum among three group was detected by Western blot. (**B**) Quantitative analysis of Western blot was performed using One-way ANOVA followed by Tukey’s multiple comparisons test. n.s. no significance versus Sham group; n.s. no significance versus Vehicle group. N=5 per group.

**
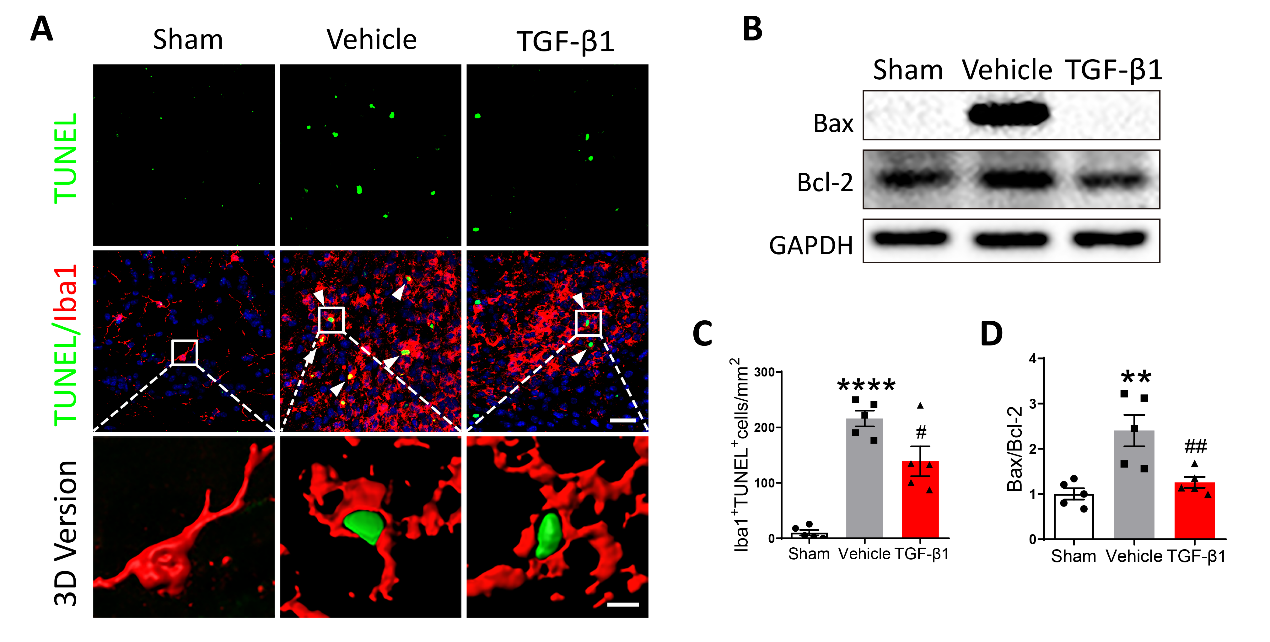
**

**Fig. S2 Preventative administration of TGF-β1 attenuated the apoptosis of microglia in LPC-modeling mice.**

(**A**) Representative confocal images of Iba1 and TUNEL in the corpus callosum among three groups, scale bar=30 μm. White arrowhead pointed to the immunofluorescent double-labeling cells. 3D reconstruction of local enlarged image was presented, scale bar=5 μm. (**B**) The protein expression of Bax and Bcl-2 in the corpus callosum among three group was detected by Western blot. (**C**) Quantitative analysis of the number of Iba1^+^TUNEL^+^cells in immunostaining. One-way ANOVA was applied for statistical analysis followed by Tukey’s multiple comparisons test. ****P<0.0001 versus Sham group; #P<0.05 versus Vehicle group. N=5 per group. (**D**) Quantitative analysis of Western blot was performed using One-way ANOVA followed by Tukey’s multiple comparisons test. **P < 0.01 versus Sham group; ##P<0.01 versus Vehicle group. N= 5 per group.

**
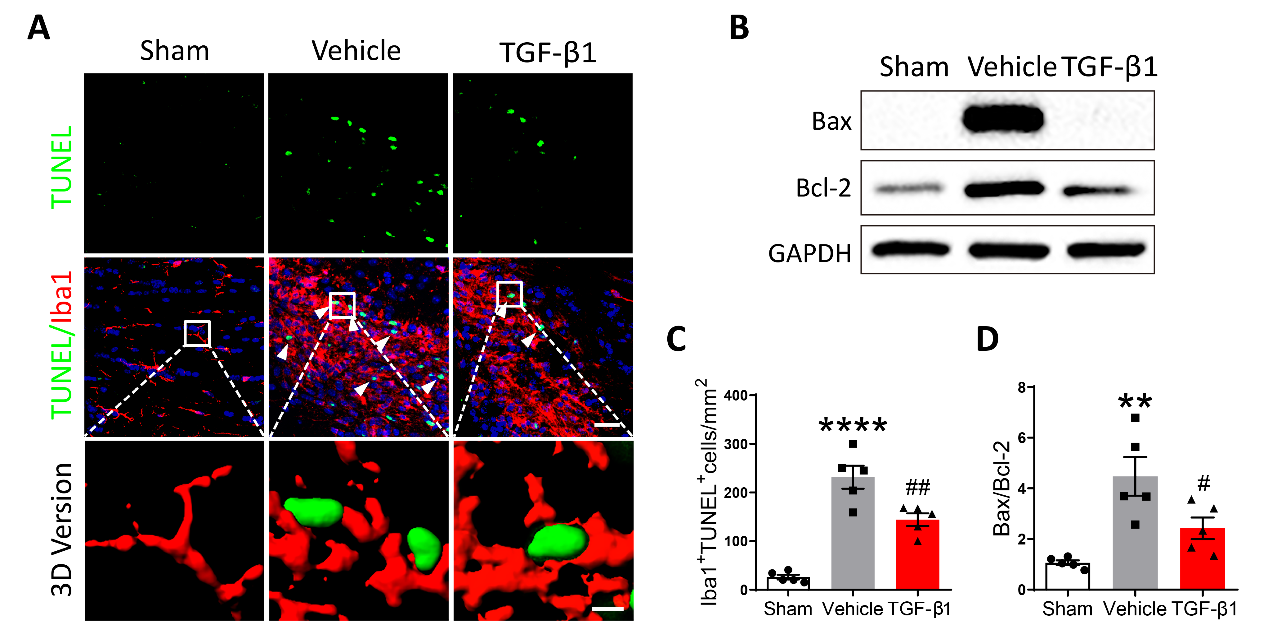
**

**Fig. S3 Therapeutic application of TGF-β1 alleviated the apoptosis of microglia in LPC-modeling mice.**

(**A**) Representative confocal images of Iba1 and TUNEL in the corpus callosum among three groups, scale bar=30 μm. White arrowhead pointed to the immunofluorescent double-labeling cells. 3D reconstruction of local enlarged image was presented, scale bar=5 μm. (**B**) The protein level of Bax and Bcl-2 in the corpus callosum among different groups was detected by Western blot. (**C**) Quantitative analysis of the number of Iba1^+^TUNEL^+^cells in immunostaining. One-way ANOVA was applied for statistical analysis followed by Tukey’s multiple comparisons test. ****P<0.0001 versus Sham group; ##P<0.01 versus Vehicle group. N=5 per group. (**D**) Quantitative analysis of Western blot was conducted using One-way ANOVA followed by Tukey’s multiple comparisons test. **P<0.01 versus Sham group; #P<0.05 versus Vehicle group. N=5 per group.


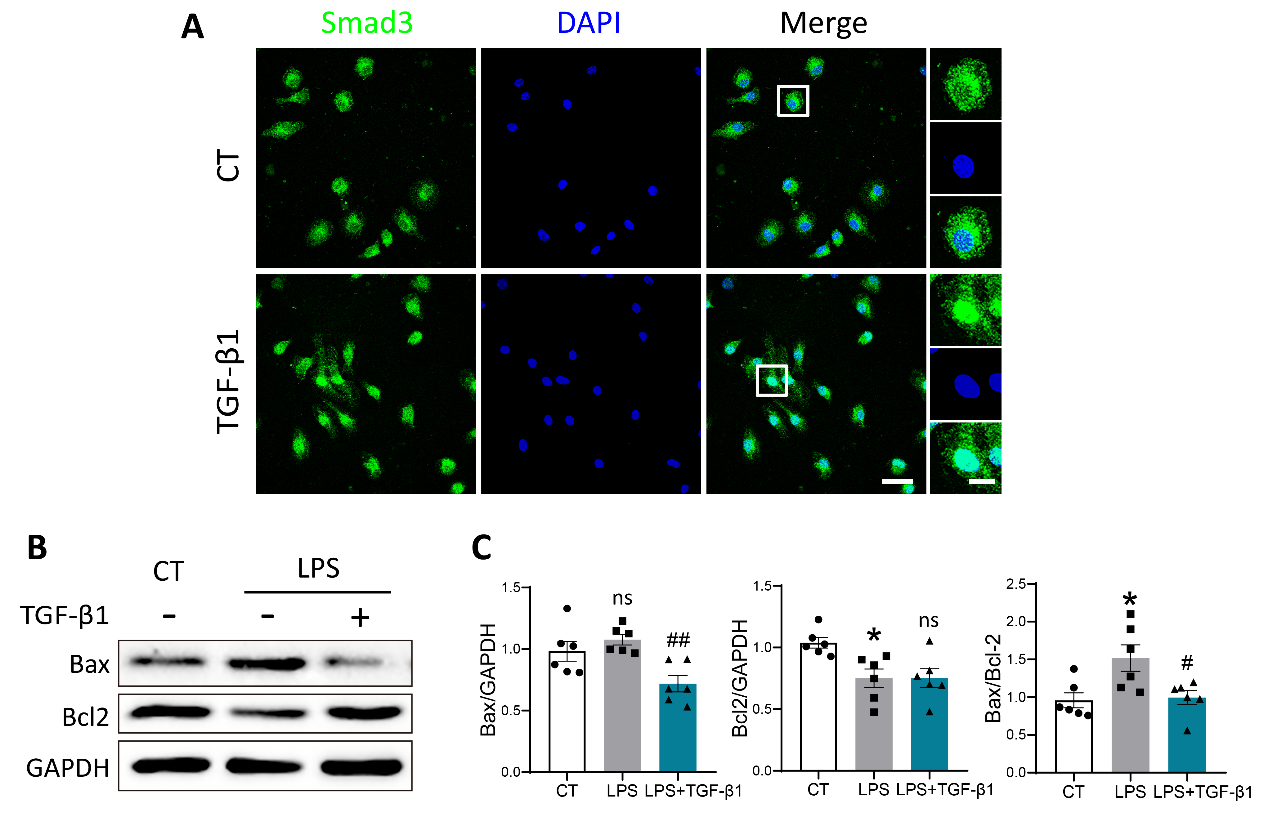


**Fig. S4 TGF-β1 attenuated the apoptotic process of primary cultured microglia stimulated by LPS *in vitro*.**

(**A**) Representative confocal images showing the translocation of Smad3 into nuclei under treatment of TGF-β1 in cultured microglia, scale bar=30 μm. Local enlarged views were demonstrated, scale bar=10 μm. (**B**) The protein expression of apoptosis-associated markers (Bax and Bcl-2) in cultured microglia was detected by Western blot. (**C**) Quantitative analysis of Western blot was conducted using One-way ANOVA followed by Tukey’s multiple comparisons test. *P < 0.05, n.s. no significance versus CT group; #P < 0.05, ##P < 0.01, n.s. no significance versus LPS group. N=6 per group.


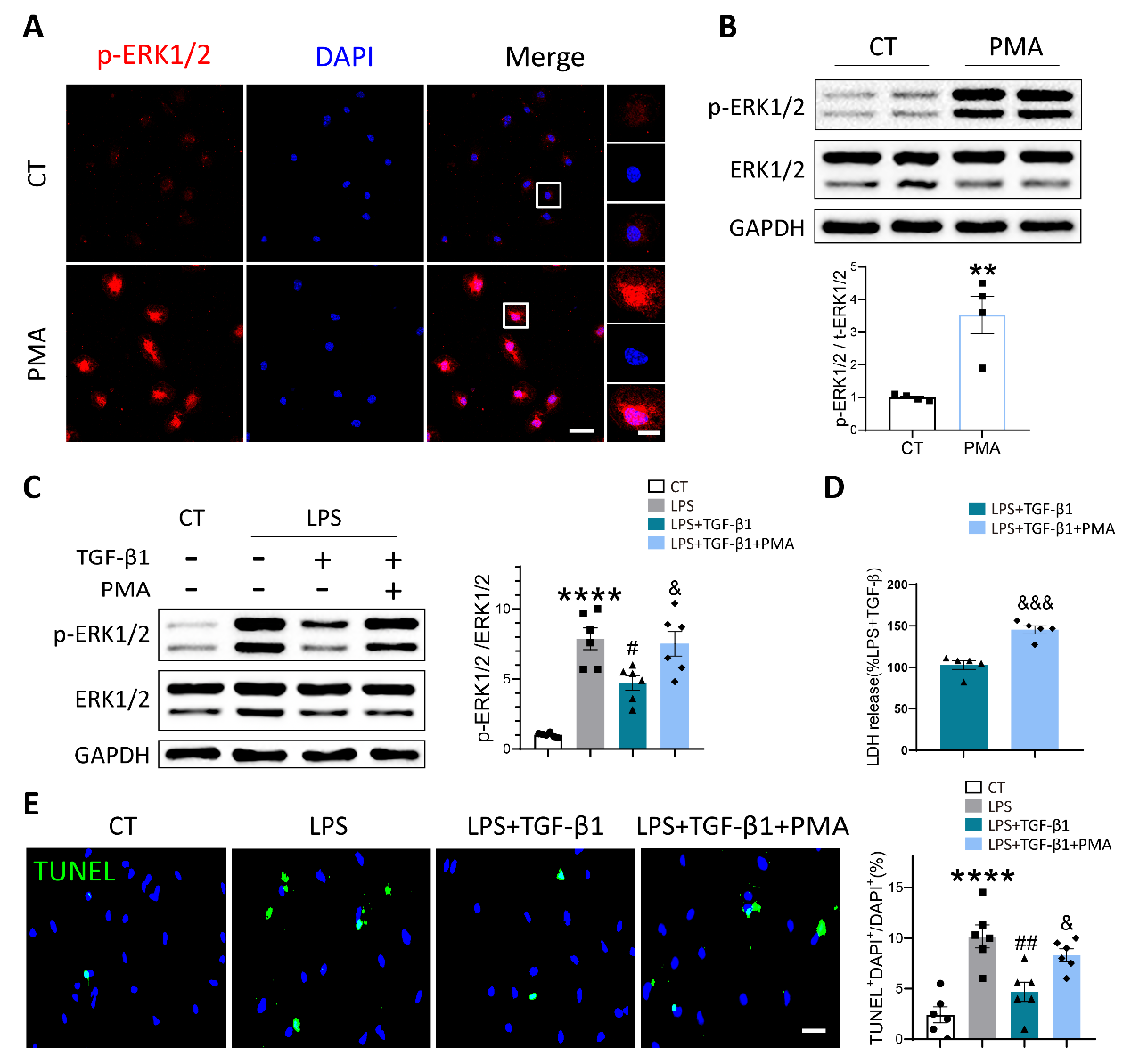


**Fig. S5 Activation of ERK1/2 and NF-κB pathways by PMA partially abolished the anti-apoptotic effect of TGF-β1 *in vitro*.**

(**A**) Representative confocal images showing the increased fluorescent intensity of p-ERK1/2 in nuclei upon addition of PMA in cultured microglia, scale bar=30 μm. Local enlarged views were demonstrated, scale bar=10 μm. (**B**) The protein expression of p-ERK1/2 and ERK1/2 in cultured microglia was assessed by Western blot. N=4 per group. Quantitative analysis of Western blot was performed using unpaired Student’s t-test. **P < 0.01 versus CT group. (**C**) The protein expression of p-ERK1/2 and ERK1/2 in cultured microglia among different groups was assessed by Western blot. N=6 per group. Quantitative analysis of Western blot was conducted using One-way ANOVA followed by Tukey’s multiple comparisons test. ****P<0.0001 versus CT group; #P<0.05 versus LPS group; &P<0.05 versus LPS+TGF-β1 group. (**D**) The LDH test of cultured microglia exposed to different treatments. Unpaired Student’s t-test was used for statistical analysis. &&&P<0.001 versus LPS+TGF-β1 group. N=5 per group.

(**E**) Representative confocal images of TUNEL staining in cultured microglia exposed to varied treatments, scale bar = 30 μm. The percentages of TUNEL^+^cells among varied groups were compared using One-way ANOVA statistical analysis followed by Tukey’s multiple comparisons. N=6 per group.
