# Supplementary material for: Transforming growth factor-β1 protects against LPC-induced cognitive deficit by attenuating pyroptosis of microglia via NF-κB/ERK1/2 pathways
Source: J Neuroinflammation. 2022 Jul 28;19:194. doi: 10.1186/s12974-022-02557-0 (PMC9336072; doi:10.1186/s12974-022-02557-0)
Supplement: Supplementary file 2 — Additional file 2. R codes for Principal Component Analysis (PCA) and heatmap in TreadScan test. [file 12974_2022_2557_MOESM2_ESM.docx]

**Additional file 2**

**R codes for clustering heatmap in TreadScan test**

filepath<-file.choose()

filepath

df<-read.csv(filepath,header=T,row.names = 1)

head(df)

library(pheatmap)

help(package="pheatmap")

df1<-as.matrix(df)

pheatmap(df1,

cluster_cols = F,

cluster_rows = T,

scale="row",

color=colorRampPalette(c("navy","white","firebrick"))(50),

cellwith=15,

cellheight=15,

treeheight_row=30)

**R codes for Principal Component Analysis (PCA) in TreadScan test**

filepath<-file.choose()

filepath

df<-read.csv(filepath,header=T,row.names = 1)

head(df)

butai<-prcomp(df[,1:81],scale=T)

summary(butai)

library(factoextra)

fviz_eig(butai,addlabels = T)

names(butai)

butai$x

butai$rotation

head(butai)

fviz_pca_ind(butai,col.ind=df$Species,

addEllipses = T,geom=("point"))
