# Supplementary material for: Transforming growth factor-β1 protects against LPC-induced cognitive deficit by attenuating pyroptosis of microglia via NF-κB/ERK1/2 pathways
Source: J Neuroinflammation. 2022 Jul 28;19:194. doi: 10.1186/s12974-022-02557-0 (PMC9336072; doi:10.1186/s12974-022-02557-0)
Supplement: Supplementary file 3 — Additional file 3. Information about antibodies applied in IF and WB. [file 12974_2022_2557_MOESM3_ESM.docx]

**Additional file 3**

**Information about antibodies applied in IF and WB**

| **Antibody** | **Manufacturer** | **Catalog** | **Host species** | **Application** |
| --- | --- | --- | --- | --- |
| **TGF-β1** | Abcam, USA | ab215715 | rabbit | IF 1:100 |
| **MBP** | Millipore, USA | MAB386 | rat | IF 1:200 |
| **Iba1** | Wako, Japan | 011-27991 | goat | IF 1:400 |
| **Iba1** | Wako, Japan | 019-19741 | rabbit | IF 1:400 |
| **CD68** | Bio-Rad, USA | MCA1957GA | rat | IF 1:400 |
| **GSDMD** | Abcam, USA | ab219800 | rabbit | IF 1:100 |
| **GSDMD** | Abcam, USA | ab209845 | rabbit | WB 1:1000 |
| **ASC** | Cell Signaling Technology, USA | 67824S | rabbit | IF 1:400  WB 1:1000 |
| **IL-1β** | Abcam, USA | ab9722 | rabbit | IF 1:100 |
| **IL-1β** | ABclonal, China | A17361 | rabbit | WB 1:1000 |
| **Smad3** | Cell Signaling Technology, USA | 9523S | rabbit | IF 1:400 |
| **NF-κB** | Cell Signaling Technology, USA | 8242T | rabbit | IF 1:400  WB 1:1000 |
| **p-NF-κB** | Cell Signaling Technology, USA | 3033T | rabbit | WB 1:1000 |
| **ERK1/2** | Cell Signaling Technology, USA | 4695T | rabbit | WB 1:1000 |
| **p-ERK1/2** | Cell Signaling Technology, USA | 4370T | rabbit | IF 1:400 |
| **NLRP3** | Adipogen, USA | AG-20B-0014-C100 | mouse | IF 1:400 |
| **NLRP3** | Cell Signaling Technology, USA | 15101S | rabbit | WB 1:1000 |
| **Caspase-1-p20** | Adipogen, USA | AG-20B-0042-C100 | mouse | WB 1:1000 |
| **Bax** | Santa, USA | sc-7480 | mouse | WB 1:1000 |
| **Bcl-2** | Abcam, USA | ab182858 | rabbit | WB 1:1000 |
| **iNOS** | ABclonal, China | A0312 | rabbit | WB 1:1000 |
| **CD206** | R&D system, USA | AF2535 | goat | WB 1:1000 |
| **GAPDH** | BOSTER, China | BM1623 | Rabbit | WB 1:1000 |
