# Supplementary material for: Transforming growth factor-β1 protects against LPC-induced cognitive deficit by attenuating pyroptosis of microglia via NF-κB/ERK1/2 pathways
Source: J Neuroinflammation. 2022 Jul 28;19:194. doi: 10.1186/s12974-022-02557-0 (PMC9336072; doi:10.1186/s12974-022-02557-0)

**Additional file 4**

**Raw images of Western blot**

**Figure 3C**

GSDMD Cleaved-GSDMD


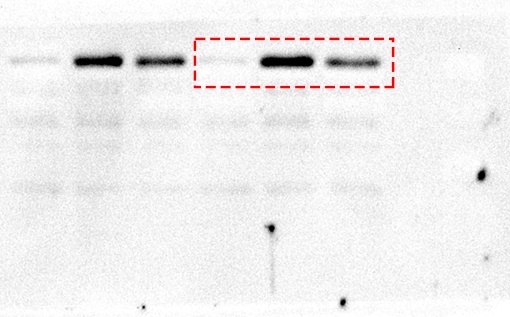

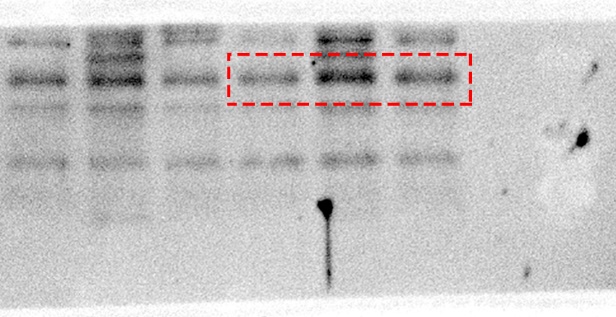


GAPDH


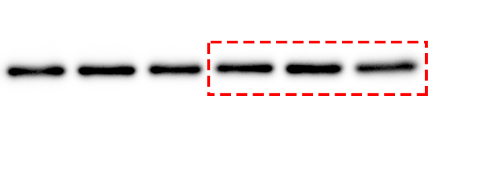


**Figure 3G**

Pro-Caspase1 Caspase1-p20


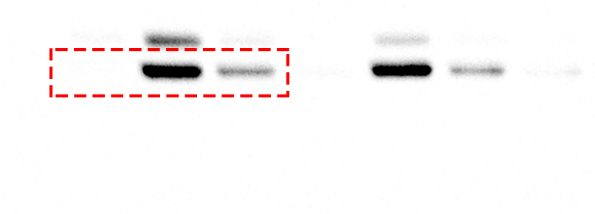

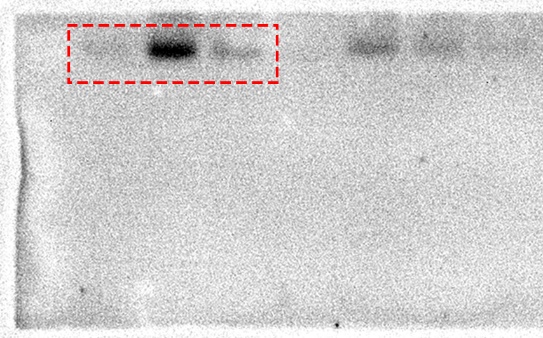


GAPDH


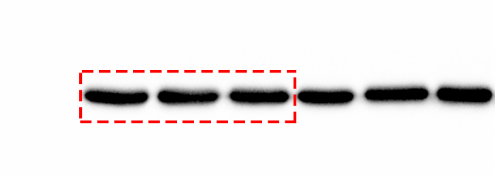


NLRP3 ASC


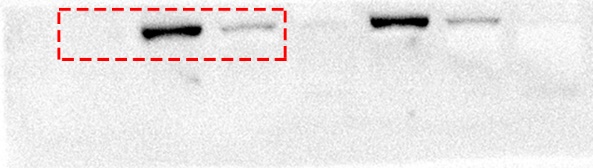

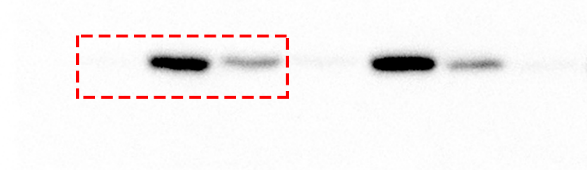


Pro-IL-1β+Cleaved- IL-1β GAPDH


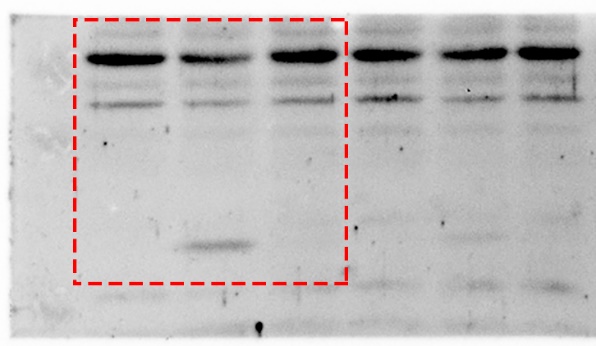

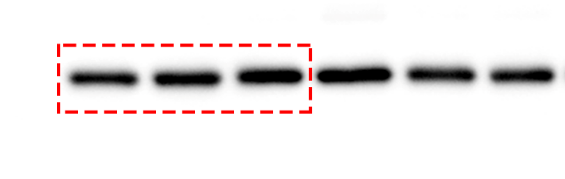


**Figure 5C**

GSDMD Cleaved-GSDMD

**
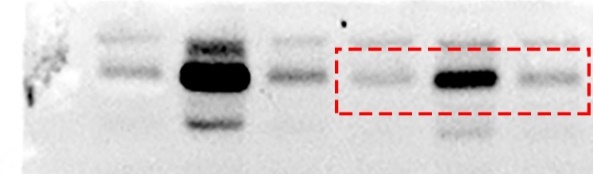

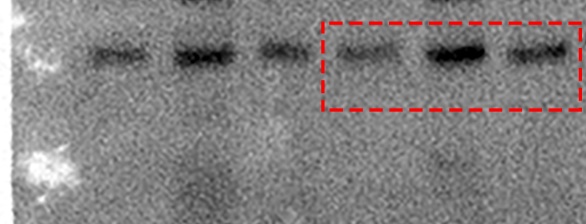
**

GAPDH


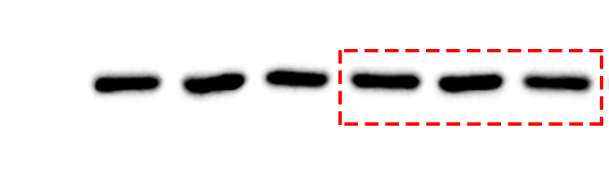


Pro-Caspase1+Caspase1-p20 NLRP3


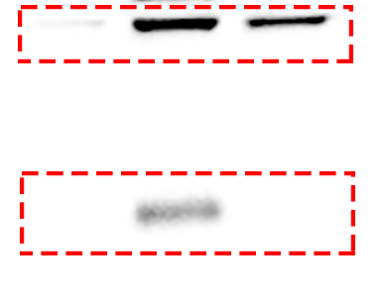

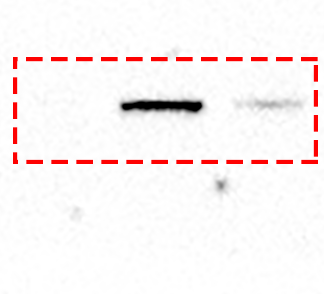


GAPDH


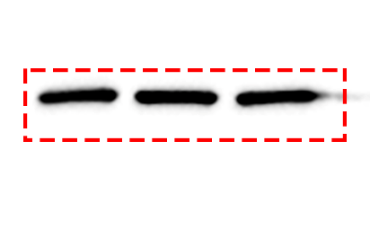


ASC Pro-IL-1β+Cleaved- IL-1β GAPDH

**
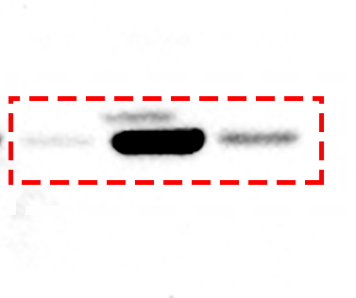

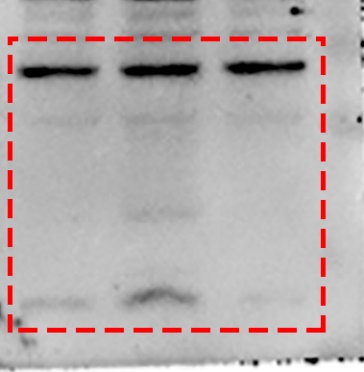

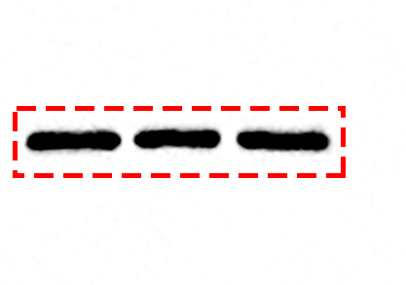
**

**Figure 6C**

NLRP3 Caspase1-p20

**
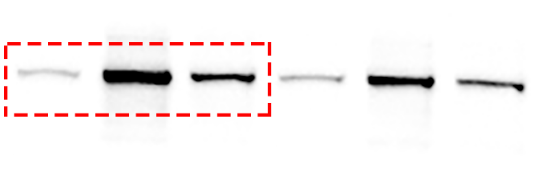
**
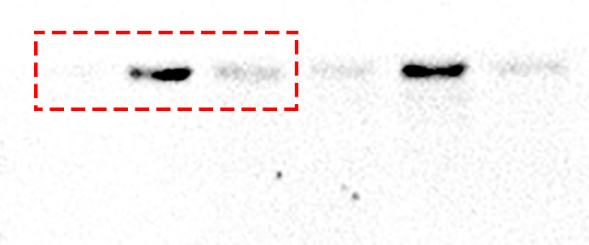


IL-1β GAPDH
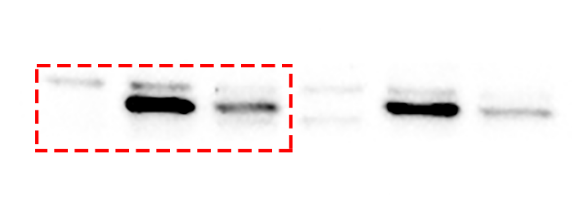
 **
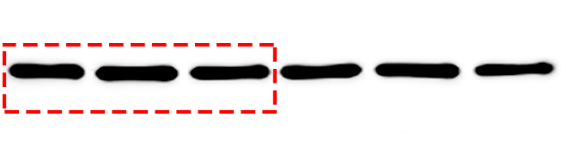
**

Cleaved-GSDMD GAPDH


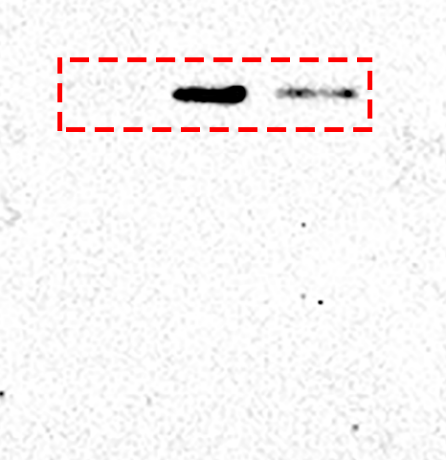

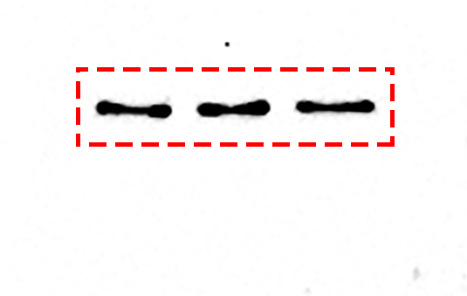


**Figure 6G**

p-NF-κB NF-κB


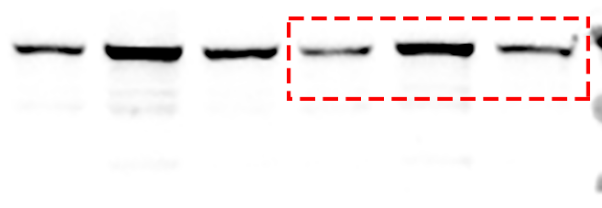

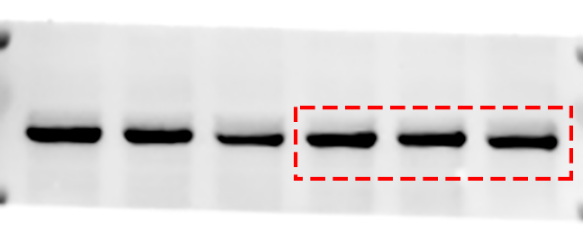


p-ERK1/2 ERK1/2


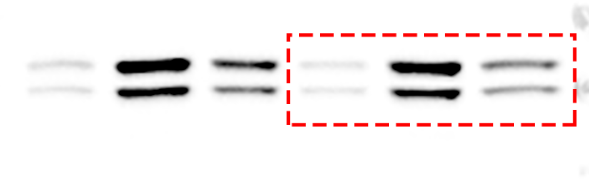

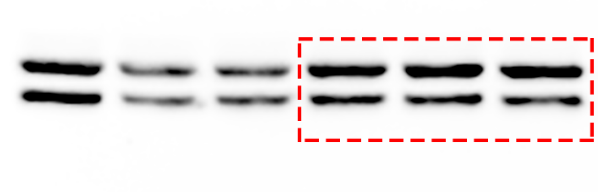


GAPDH


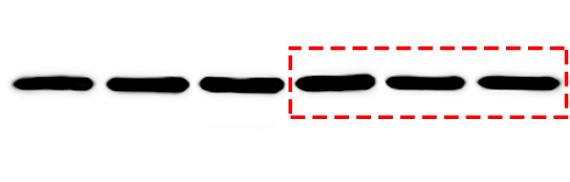


**Figure 7A**

NLRP3 IL-1β


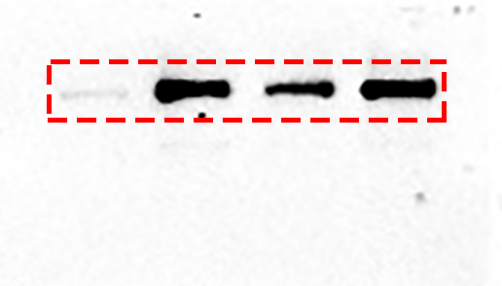

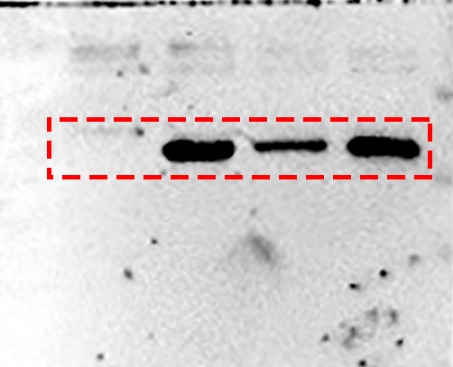


Cleaved-GSDMD GAPDH


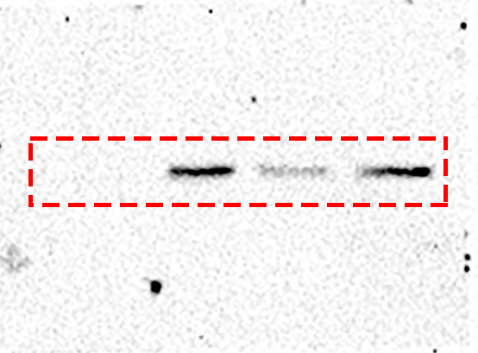

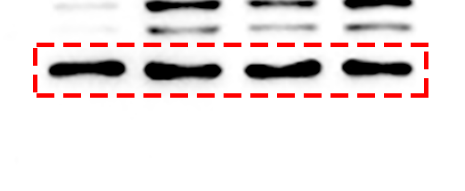


**Figure S1A**

iNOS CD206


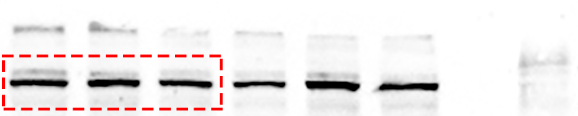

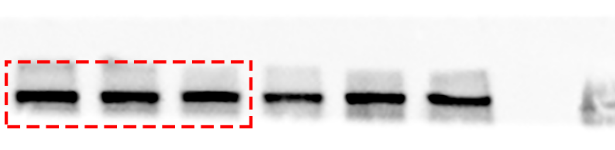


GAPDH


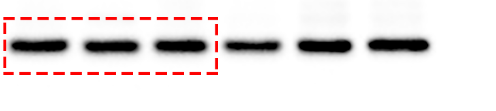


**Figure S2B**

Bax Bcl2


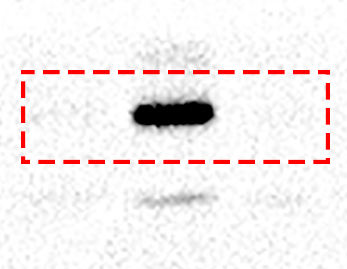

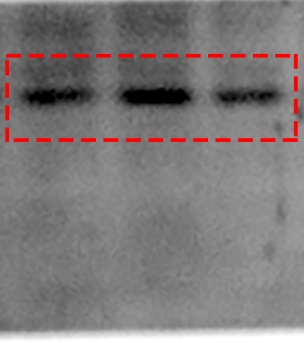


GAPDH


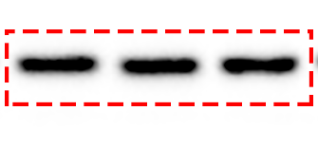


**Figure S3B**

Bax Bcl2


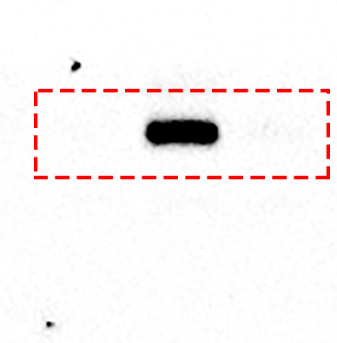

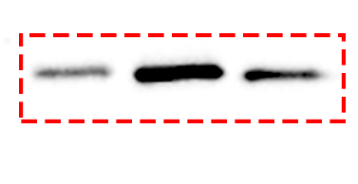


GAPDH


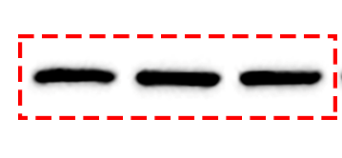


**Figure S4B**

Bax Bcl2


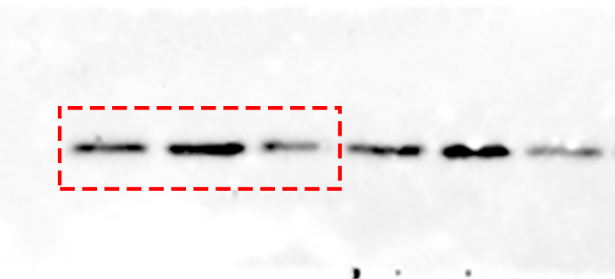

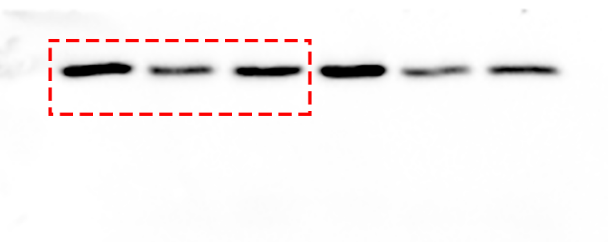


GAPDH


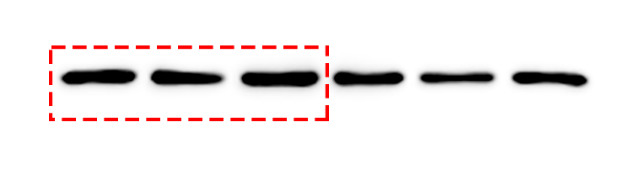


**Figure S5B**

p-ERK1/2 ERK1/2


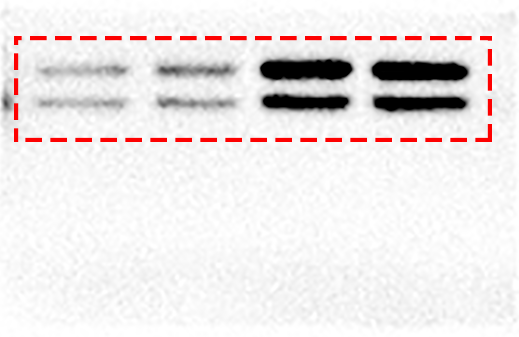

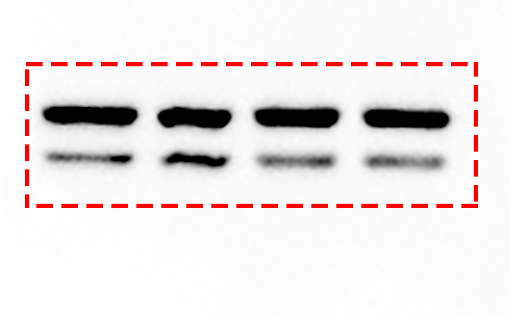


GAPDH


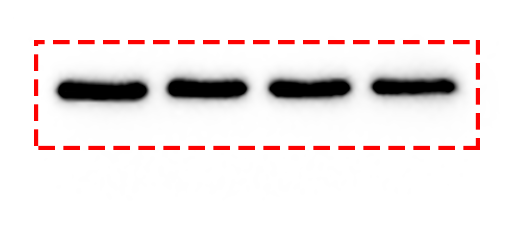


**Figure S5C**

p-ERK1/2 ERK1/2

**
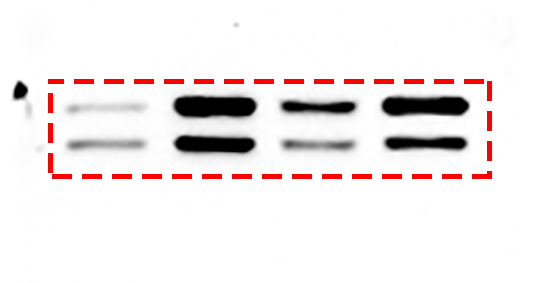

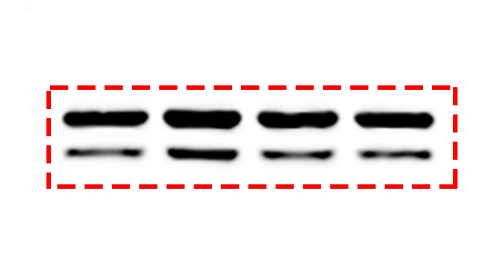
**

GAPDH


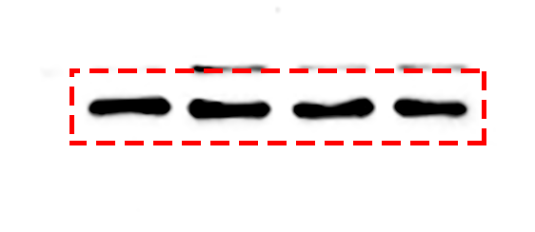

Supplement: Supplementary file 4 — Additional file 4. Raw images of Western blot. [file 12974_2022_2557_MOESM4_ESM.docx]
